# Tobacco smoke exposure is a driver of altered oxidative stress response and immunity in head and neck cancer

**DOI:** 10.1186/s12967-025-06258-z

**Published:** 2025-04-05

**Authors:** Yang Li, Pedram Yadollahi, Fonma N. Essien, Vasanta Putluri, Chandra Shekar R. Ambati, Karthik Reddy Kami Reddy, Abu Hena Mostafa Kamal, Nagireddy Putluri, Lama M. Abdurrahman, Maria E. Ruiz Echartea, Keenan J. Ernste, Akshar J. Trivedi, Jonathan Vazquez-Perez, William H. Hudson, William K. Decker, Rutulkumar Patel, Abdullah. A. Osman, Farrah Kheradmand, Stephen Y. Lai, Jeffrey N. Myers, Heath D. Skinner, Cristian Coarfa, Kwangwon Lee, Antrix Jain, Anna Malovannaya, Mitchell J. Frederick, Vlad C. Sandulache

**Affiliations:** 1 Bobby R. Alford Department of Otolaryngology Head and Neck Surgery, Baylor College of Medicine, One Baylor Plaza, MS: NA102, Houston, TX 77030 USA; 2 Advanced Technology Cores, Dan Duncan Cancer Center, Baylor College of Medicine, Houston, TX USA; 3 Dan L Duncan Comprehensive Cancer Center, Baylor College of Medicine, Houston, TX USA; 4 Department of Molecular and Cellular Biology, Baylor College of Medicine, Houston, TX USA; 5 Center for Precision Environmental Health, Baylor College of Medicine, Houston, TX USA; 6 Department of Pathology and Immunology, Baylor College of Medicine, Houston, TX USA; 7 Center for Cell Gene Therapy, Baylor College of Medicine, Houston, TX USA; 8 Department of Radiation Oncology, Baylor College of Medicine, Houston, TX USA; 9 Department of Head and Neck Surgery, University of Texas MD Anderson Cancer Center, Houston, TX USA; 10 Department of Medicine-Pulmonary, Baylor College of Medicine, Houston, TX USA; 11 Center for Translational Research on Inflammatory Diseases, Michael E. DeBakey Veterans Affairs Medical Center, Houston, TX USA; 12 Department of Radiation Oncology, University of Pittsburgh, Pittsburgh, PA USA; 13 Verna and Marrs Mclean Department of Biochemistry and Molecular Pharmacology, Baylor College of Medicine, Houston, TX USA; 14 Mass Spectrometry Proteomics Core, Baylor College of Medicine, Houston, TX USA; 15 Department of Molecular and Cellular Oncology, University of Texas MD Anderson Cancer Center, Houston, USA; 16 Department of Radiation Oncology, University of Texas MD Anderson Cancer Center, Houston, USA

**Keywords:** Tobacco, Nrf2, Oxidative stress, Keap1, Glutathione, PDL1

## Abstract

**Background:**

Exposomes are critical drivers of carcinogenesis. However, how they modulate tumor behavior remains unclear. Extensive clinical data show cigarette smoke to be a key exposome that promotes aggressive tumors, higher rates of metastasis, reduced response to chemoradiotherapy, and suppressed anti-tumor immunity. We sought to determine whether smoke itself can modulate aggressive tumor behavior in head and neck squamous cell carcinoma (HNSCC) through reprogramming of the cellular reductive state.

**Methods:**

Using established human and murine HNSCC cell lines and syngeneic mouse models, we utilized conventional western blotting, steady state and flux metabolomics, RNA sequencing, quantitative proteomics and flow cytometry to analyze the impact of smoke exposure on HNSCC tumor biology and anti-tumor immunity.

**Results:**

Cigarette smoke persistently activated Nrf2 target genes essential for maintenance of the cellular reductive state and survival under conditions of increased oxidative stress in HNSCC regardless of human papillomavirus (HPV) association. In contrast to e-cigarette vapor, conventional cigarette smoke mobilizes cellular metabolism toward oxidative stress adaptation, resulting in development of cross-resistance to cisplatin. In parallel, smoke exposure modulates expression of PDL1 and the secretory phenotype of HNSCC cells resulting in an altered tumor immune microenvironment (TIME) in syngeneic mouse models and downregulated expression of antigen presentation and costimulatory genes in myeloid cells.

**Conclusion:**

The cigarette smoke exposome is a potent activator of the Nrf2 pathway and appears to be the primary trigger for a tripartite phenotype of aggressive HNSCC consisting of: (1) reduced chemotherapy sensitivity, (2) enhanced metastatic potential and (3) suppressed anti-tumor immunity.

**Supplementary Information:**

The online version contains supplementary material available at 10.1186/s12967-025-06258-z.

## Introduction

Head and neck squamous cell carcinoma (HNSCC) is the sixth most prevalent cancer worldwide and is strongly linked to tobacco use, alcohol consumption, and human papillomavirus (HPV) infection [1]. Patients with HPV-associated cancer generally experience excellent outcomes after radiotherapy or chemoradiotherapy compared to those with HPV-independent cancers [2]. However, multiple prospective and retrospective analyses have shown that in cigarette smokers the survival advantage associated with HPV-associated oropharyngeal squamous cell carcinoma (OPSCC) is significantly reduced [3–5]. Specifically, 5 year overall survival is 20–40% lower in smokers compared to non-smokers depending on HPV status [6–9].

Whereas most studies concerning tobacco smoke exposure have addressed the risk of carcinogenesis, our work has continuously focused on the impact of tobacco exposure, particularly that present at the time of cancer diagnosis and throughout treatment, on modulation of tumor biology, anti-tumor immunity and ultimately the effectiveness of chemoradiotherapy regimens [3, 4, 10, 11]. Tobacco smoke has now been linked to activation of indoleamine 2,3-dioxygenase 1 (IDO1) and immune modulation including increased infiltration of regulatory T-cells (Tregs) and reduced levels of cytotoxic T lymphocytes. [12] Furthermore, tobacco-associated carcinogens have been reported to directly induce chemoresistance through modulation of oxidative stress [13, 14]; persistent tobacco exposure can increase cellular oxidative stress, leading to continuous activation of antioxidant response element signaling [15–17].

We previously showed a link between tobacco smoke exposure and rates of chemoradiotherapy failure in HNSCC, regardless of HPV association [3, 11]. HNSCC patients with extensive tobacco smoke exposure, particularly those actively smoking at the time of diagnosis also demonstrated a suppressed, and potentially less functional anti-tumor immune response [10, 11]. Using patient-level data, and preclinical HNSCC models, we subsequently linked hyperactivation of the Nuclear factor erythroid 2-related factor 2 (Nrf2) signaling pathway with chemotherapy and radiation resistance in HNSCC [18, 19]. This occurs largely through a metabolic adaptation to higher levels of chronic oxidative stress, resulting in an enhanced reductive state (higher levels of reducing equivalents) able to reset overall redox homeostasis. In the current study, we sought to determine whether Nrf2 signaling activated through tobacco smoke exposure could provide a common mechanistic link between altered immunity and reduced effectiveness of chemoradiotherapy through modulation of the oxidative stress response and metabolic adaptation in HNSCC regardless of HPV association.

## Methods

### Cell lines

Human HPV-associated HNSCC SCC090 (RRID: CVCL_1899), SCC152 (RRID: CVCL_C058), SCC 154 (RRID: CVCL_2230), and UDSCC2 (RRID: CVCL_E325) and HPV-independent cell lines UMSCC22A (RRID: CVCL_7731), HN30 (RRID: CVCL_5525), HN31 (RRID: CVCL_5526) and UMSCC47 (RRID:CVCL 7759) were maintained in high glucose DMEM, MEM or RPMI as previously described [20, 21], supplemented with 10% fetal bovine serum, 1% glutamine, 1% vitamins, 1% non-essential amino acids, 1% penicillin/streptomycin, 1% sodium pyruvate, and 0.2% MycoZap™ Prophylactic (LT07-318, Lonza) to prevent mycoplasma overgrowth. Mouse MOC1 cells (RRID: CVCL_ZD32) were cultured as previously described [22].

Genetically engineered mouse models (GEMMs) of HPV-negative HNSCC were created using the Cre-LoxP system to induce tissue-specific alterations of genes, such as *TP53* and *PIK3CA* with or without *KEAP1*. These genes are among the most frequently altered in human HNSCC. Keap1-proficient (*KEAP1*^+/+^) and Keap1-deficient (*KEAP1*^fl/fl^) primary tumors isolated from GEMMs were digested using a Miltenyi tumor dissociation kit (Cat # 130-096-730), and a single-cell suspension was generated. *KEAP1*^+/+^ and *KEAP1*^fl/fl^ clones A, D, E, and F were provided by Dr. Rutulkumar Patel. *KEAP1*^+/+^ cell lines have intact copies of *KEAP1*, loss of both copies of *TP53* and harbor an activating *PIK3CA mutation (H1074R)*. *KEAP1*^fl/fl^ cell lines have one *KEAP1* copy deleted and the other floxed, as well as loss of both copies of *TP53* and activation of oncogenic *PIK3CA*. Clones A, D, E, and F were isolated from the *KEAP1*^l fl/fl^ cells. Cell lines were cultured at 37 °C in a humidified incubator with 5% CO_2_. Cisplatin resistant polyclonal populations utilized were previously described [23]. Mycoplasma-free conditions were confirmed using the MycoAlert mycoplasma detection kit (Lonza, Basel, Switzerland). Cell line origin was confirmed by short tandem repeat (STR) analysis every 3 months.

Smoke-infused media was freshly prepared before each experiment by drawing smoke from 1 cigarette into 15 mL of MEM over 1 min, followed by sterile filtration. Cells were treated with the indicated concentration of smoke-infused media for the indicated times in acute smoke exposure settings. Chronic smoke exposure consisted of repeated exposure to increasing fractions of smoke-infused media from 0.5 to 4% depending on the cell line over a 6-month period, with application between 1 and 3 times per week. Electronic cigarette vapor exposures were generated using 1.2 mL of liquid vape (nicotine concentration of 1.2 mg/mL, comparable to the nicotine content in one Marlboro Red cigarette) vaporized into 15 mL MEM medium.

### Western blotting

Cells were subjected to western blot analysis as previously reported [22] using the following antibodies: Nrf2 (PA5-27882, Invitrogen, RRID:AB_2545358), Keap1 (8407, Cell Signaling, RRID:AB_10860776), NF-κB P65 (sc-8008, Santa Cruz Biotechnology, RRID:AB_628017), Gpx2 (MA5-36260, Invitrogen, RRID:AB_2890391), Nqo1 (ZRB1707, Millipore Sigma, RRID:AB_3662860), β-actin (sc-81178, Santa Cruz Biotechnology, RRID:AB_2223230). NE-PER Nuclear and Cytoplasmic Extraction Kit (78,833, Invitrogen) was used to isolate cytosolic and nuclear fractions according to the manufacturer’s instruction followed by the western blot analysis. Histone H3 (4499, Cell Signaling, RRID: AB_10544537) was used as a nuclear fraction loading control, and α-tubulin (ab7291, Abcam) as the cytosolic fraction loading control.

### Generation of *NFE2L2 *(i.e., NRF2) and *KEAP1* knockdown stable cell lines

GIPZ lentiviral human *NFE2L2* shRNA glycerol stocks (Clone Id: V2LHS_239104, V2LHS_64253), lentiviral human *KEAP1* shRNA glycerol stocks (Clone Id: V2LHS_254870, V3LHS_344984) and an empty vector (EV) control were obtained from Horizon Discovery. Lentiviruses were generated by transfecting HEK293T cells with shRNA or EV, envelope plasmid pMD2.G (#12259, Addgene, RRID: Addgene_12259) and the packaging vector psPAX2 (#12260, Addgene, RRID: Addgene_12260). Cells were infected with either an EV, *NFE2L2* shRNA or *KEAP1* shRNA and selected for effective infection using 4 µg/mL puromycin; protein levels were confirmed by immunoblotting.

### Measurement of cellular redox states

Cells were labeled with 20 µM DCFDA (ab113851, Abcam) for 30 min and then treated with various concentrations of smoke-infused media for 1 h. Cellular ROS levels were measured by fluorescence activated cell sorting (FACS) using a BD FACS Aria flow cytometer.

### Cell cycle distribution analysis

Cells were seeded in 6cm dishes and treated with various concentrations of smoke the following day for 24 h and 48 h. Cells were harvested and fixed overnight in 70% ethanol at 4 °C. They were then stained with 20 µg/mL propidium iodide and 100 µg/mL RNase for 1 h at 37 °C, followed by analysis using a BD FACS Aria flow cytometer.

### FITC Annexin V apoptosis detection

Cells were seeded in 6 cm dishes and treated with various concentrations of smoke the following day for 48 h. After treatment, cells were harvested and incubated in a buffer containing FITC Annexin V and propidium iodide (556547, BD Pharmingen™) for 15 min at room temperature (25 °C) in the dark, followed by flow cytometric analysis.

### RNA-seq and ssGSEA analysis of TCGA cohort and cell lines

UMSCC47 (RRID: CVCL_7759) and UDSCC2 cells were harvested after acute exposure to smoke-infused media for 8 h, whereas SCC152 and SCC 154 cells were harvested following chronic smoke-infused media exposure with an additional bolus 8 h prior to isolation. Total RNA from biological replicates was extracted using the RNeasy Mini Kit (74104, Qiagen) according to the manufacturer’s instructions and analyzed by whole transcriptomic sequencing (Psomagen Inc), which returned RSEM counts and effective gene sizes. Samples were normalized by calculating the FPKM-UQ values and then log transformed as log2 (X + 0.01) for statistical analysis, except for single sample gene set enrichment analysis (ssGSEA) which was performed on data in linear space using the Broad Institute’s Gene Pattern website platform. Previously harmonized RNA-seq RSEM data from TCGA were downloaded from the Toil open-source data portal and normalized to derive FPKM-UQ values as we previously described [22]. For differential analysis of gene expression, only genes with an average log 2 FPKM value ≥ 2 for at least one group (i.e., treatment or control) were considered to avoid genes with low expression. Differentially expressed genes between smoke treated  and sham treated cells were analyzed with JMP 13 statistical software, which performs individual t tests for each gene and applies a Benjamini–Hochberg correction (FDR < 0.1) to calculate adjusted p values. Fold-changes in gene expression refer to the ratio of geometric means (2^[log fold change]. Gene lists used for leukocyte and Nrf2 pathway ssGSEA score calculations were previously published and validated [22]. Essentially, cross-correlation coefficients of gene expression data were analyzed by two-way hierarchical clustering to identify modules of like expression, which were then validated by comparison with independent data or an orthogonal approach [22, 24].

### Consensus hierarchical clustering

We previously described our in-house MATLAB script to perform consensus hierarchical clustering based on Ward’s linkage [22, 24], which uses a modification of the resampling-based method published by Mont et al. Samples are clustered using Z scores derived independently for each gene (e.g., sample-wise Z score within a gene) and the already generated Z scores are transposed and re-used when clustering by gene. Matlab script is publicly available at https://github.com/aif33/Hierarchical-two-way-agglomerative-consensus-clustering.

### Proteomics

Cells were exposed to cigarette smoke-infused media for various periods and subjected to protein analysis. Protein extraction, digestion, and peptide fractionation were performed as previously described [25]. Briefly, cells were lysed in 8 M urea buffer, reduced and alkylated, and digested with LysC and Trypsin proteases. Twenty micrograms of peptide per sample was labeled with TMT16 pro plex isobaric label reagent (Thermo Fisher Scientific) according to the manufacturer’s protocol. High-pH offline fractionation was carried out to generate 24 peptide pools, which were acidified with a final concentration of 0.1% formic acid (FA). The deep-fractionated peptide samples were separated on a nano-LC 1200 system (Thermo Fisher Scientific, San Jose, CA) coupled to an Orbitrap Lumos ETD mass spectrometer (Thermo Fisher Scientific, San Jose, CA). The samples were loaded onto a 2 cm × 100 µm I.D. switched in-line with a 20 cm × 75 µm I.D. separation column (Reprosil-Pur Basic C18, 1.9 µm, Dr. Maisch GmbH, Germany) and equilibrated in 0.1% formic acid/water. The column temperature was maintained at 60^o^C. Peptide elution was performed using a 110 min discontinuous gradient of 90% acetonitrile buffer (B) in 0.1%formic acid at 250 nl/min (2–35%B, 86 min, 35–60%B, 6 min, 60–95%B, 9 min, 95–50%B, 9 min). The eluted peptides were directly electro-sprayed into a mass spectrometer operated in the data-dependent acquisition mode, acquiring HCD fragmentation spectra for a 2 s cycle time. MS1 was performed in Orbitrap (120,000 resolution, scan range 375–1500 m/z, 50 ms Injection time), followed by MS2 in Orbitrap at 30,000 resolution (HCD 38%) with the TurboTMT algorithm. Dynamic exclusion was set to 20 s, and the isolation width was set to 0.7 m/z. Raw MS data processing, quantification, and differential analysis were performed as described previously [26]. Reverse decoys and common contaminants were added to the NCBI human protein database (downloaded 2020_03_24) using Philosopher [27]. Proteomics raw data files were converted to mzML using the MSConvert software. MASIC [28] was used to extract the precursor ion intensities for each peptide using the area under the elution curve as well as the reporter ion intensities. A smoothing method was used with a sampling frequency of 0.25 and an SCI tolerance of 10 ppm. Reporter ion tolerance was set to 0.003 Da, with reporter ion abundance correction enabled. Raw spectra were searched using MSFragger (v3.5) [29] and run with mass calibration. Settings included strict trypsin digestion for amino acids from 7 to 50 amino acids with up to two missed cleavages, clip protein N-term methionine, precursor ions with charge 2–6, precursor mass mode set to CORRECTED, and isotope error set to − 1/0/1/2. For fragment spectra, TopN was set to 150, and the mass range was cleared between 125 and 135 to filter out reporter ions during spectral matching. The precursor mass error was set at ± 20 ppm, and a fragment mass tolerance of ± 0.02 Da. Analyses were adjusted for static modification of C + 57 carbamidomethylation and peptide N-terminus TMT16 label: + 304.207. Dynamic modifications were set for methionine oxidation (+ 15.9949), acetylation (+ 42.0106), peptide N-terminal TMT16 label (+ 304.207), peptide N-terminal acetylation, and peptide N-terminal pyroGlu (− 17.02650 for Q, C), and (− 18.01060 for E). Multiple variable modifications were applied. Peptide validation was performed using a semi-supervised learning procedure in Percolator, as implemented by MokaPot [30]. Peptides were grouped and quantified into gene product groups using gpGrouper [31]. The resulting protein values were median-normalized and log-transformed. Heatmaps were generated using ComplexHeatmap [32]. Data wrangling was performed with Python 3.8 (Python Software Foundation. Python Language Reference http://www.python.org), along with the third-party libraries Numpy31 and Pandas (mckinney-proc-scipy-2010, reback2020pandas). For proteomics data, group differences were assessed using the moderated t test, as implemented in the R package limma (R version 4.2), and the false discovery rate was controlled using the Benjamini–Hochberg procedure.

### Steady state and ^13^C flux metabolomics

Targeted steady-state metabolomic profiling was performed on cells exposed to smoke-infused media as previously described [19, 33]. To analyze the metabolic flux with [U-^13^C]-glucose, SCC1522 and UDSCC2 cells were plated in 10 cm dishes overnight. Subsequently, they were incubated with media containing 25 mM [U-^13^C]-glucose in 0%, 5%, and 15% cigarette smoke-infused media for 72 h. Following incubation, the medium was aspirated and the cells were washed three times with cold PBS. Equal numbers of cells were harvested, snap-frozen in liquid nitrogen, and stored at −80 °C until metabolite extraction. [U-^13^C]-glucose-labeled cells were subjected to freeze–thaw cycles in liquid nitrogen, followed by homogenization in methanol: water (1:1) using needle sonication. The resulting samples were centrifuged for 10 min at 4 °C (5000 rpm). Subsequently, the samples were filtered through a 3 K Amicon filter to eliminate proteins and lipids, dried using a speed vacuum, and reconstituted with a mixture of methanol and water (1:1). Glycolysis, TCA, and PPP intermediates and isotopomers were quantified using a Luna NH2 column (3 µm, 150 × 2 mm, Phenomenex, Torrance, CA) following a previously described method [33, 34]. For cysteine flux, the amino acid metabolites were separated through the XBridge Amide HPLC column (3.5 µm, 4.6 × 100 mm, Waters, Milford, MA) in both ESI positive (Method A). For ESI in positive mode, mobile phase A consisted of 0.1% formic acid in water, while mobile phase B consisted of 0.1% formic acid in  acetonitrile. Gradient flow: 0–3 min 85% B; 3–12 min 30% B, 12–15 min 2% B, 16 min 95% B, followed by re-equilibration till the end of the gradient 23 min to the initial starting condition of 85% B. Flow rate of the solvents used for the analysis is 0.3 mL/min. The injection volume was 10 µl. Metabolite peak areas were log2 transformed and represented as a heat map of Z-scores as previously described [33].

For untargeted metabolomics, the cells were freeze-thawed and  sonicated in 50/50 methanol/water. Three volumes of methanol/acetonitrile (50/50, v/v) were then added to the samples. The samples were vortexed for 5 min and kept at − 20 °C for 10 min, then centrifuged for 10 min at 4 °C and 15,000 rpm. The samples were then evaporated to dryness using a GeneVac EZ-2 Plus SpeedVac (SP Scientific). Aliquots were reconstituted in 100 µL of methanol/water (50/50, v/v). Twenty microliters (20 µL) of each supernatant were pooled and separated into aliquots for quality control (QC). The separation was performed using a Thermo Scientific Vanquish Horizon UHPLC system. A Waters ACQUITY HSS T3 reversed phase column (1.8 μm, 2.1 mm × 150 mm) was used for reversed phase separation, and a Waters ACQUITY BEH amide hydrophilic interaction liquid chromatography (HILIC) column (1.7 μm, 2.1 mm × 150 mm) was used for separation. For the reverse phase, the gradient was from 99% mobile phase A (0.1% formic acid in water) to 95% mobile phase B (0.1% formic acid in methanol) for 21 min. For HILIC phase, the gradient was the same as that of solvent A: 0.1% formic acid, 10 mM ammonium formate, 90% acetonitrile, 10% H2O, and solvent B: 0.1% formic acid, 10 mM ammonium formate, 50% acetonitrile, and 50% H2O. For positive and negative modes of RP and HILIC, gradient flow: 0–3 min 1% B; 4–11 min 50% B, 12–21 min 95% B, followed by re-equilibration until the end of the gradient 30 min to the initial starting condition of 1% B. Both columns were run at 50 °C with a flow rate of 300 μL/min and an injection volume of 2 μL. A Thermo Scientific Orbitrap IQ-X Tribrid Mass Spectrometer was used for data collection with a spray voltage of 3500 V for the positive mode on a reverse phase separation and 2500 V for the negative mode on a HILIC phase separation using the H-ESI source. The vaporizer temperature and ion transfer tube were both 300 °C. The capillary temperature and auxiliary gas heater temperature were set at 300 and 350 °C, respectively. The sheath gas, auxiliary gas, and sweep gas flow rates were set to 40, 8, and 1 (in arbitrary units), respectively. For full MS, the data were acquired using an orbitrap detector with 120,000 resolution and quadrupole isolation. The scan range was set from 70 to 900 m/z, the normalized automatic gain control (AGC) target was 25%, and the absolute AGC value was 1.000e5 for positive and negative polarity. Compounds were fragmented by data-dependent MS/MS using HCD activation with HCD collision energies of 30, 50, and 150% via quadrupole isolation, and the detector type was orbitrap with 30,000 resolutions. The normalized automatic gain control (AGC) target was 100%, whereas the absolute AGC value was 5.000e5 for positive and negative polarity with a 50 ms maximum injection time. The acquired dataset was mined using Compound Discoverer (version 3.3.3.2, ThermoFisher Scientific, USA) for peak detection, integration, and identification. Metabolite identification was performed using the combination of in-house retention time with MS/MS database, MS/MS based mzCloud and NIST 2020 (HRMS) library, and mass accuracy based Human Metabolomic Database (HMDB) with accuracy less than 5ppm.  A pooled quality control (QC) sample was run between every 10 samples during LC/MS analysis and used as a reference sample during metabolite identification using Compound Discoverer. For data analysis, the peak area was log2 transformed, and the median IQR method was used for normalization. Differential metabolites were identified by performing a Student’s t test to calculate p values, followed by determination of the false discovery rate (FDR < 0.25) using the Benjamini–Hochberg method.

### Integrated metabolomics—transcriptomic analysis

Genes associated with differentially expressed metabolites were determined by using the Human Metabolome Database (HMDB) [35], as described previously [36, 37]. Enriched pathways based on differentially expressed metabolites were determined using over-representation analysis implemented in MSigDB [38] compared with the Hallmark and the Gene Ontology databases. A hypergeometric distribution test was used with significance defined as FDR-adjusted p value < 0.05. To integrate differentially expressed metabolites (DEMs) with differentially expressed genes (DEGs) from matching experiments, the DEGs were cross-referenced to genes associated with DEMs to create an overlapping signature.

### Hoechst assay

Hoechst assays were used to evaluate the cytotoxicity of cells exposed to smoke-infused media using established techniques [18, 19, 23, 39, 40].

### ELISAs

Cells were seeded at the same density on day 1 and changed to fresh media the following day (day 2). The conditioned media were harvested after 24 and 48 h (days 3 and 4), and the total number of cells was counted accordingly. Secreted Prostaglandin E2 (PGE2; #514010) and Interleukin-6 (IL-6; #583371, #501030) levels were measured using commercial ELISA kits (Cayman Chemical, MI USA, R&D Systems, MN, USA) and normalized to cell number. All conditions were tested in triplicate and are represented as mean ± SD. p values were calculated using Student’s t test.

### Conditioned media experiments and flow cytometry

Two million parental HN30 cells and their 4% chronic smoke exposed counterparts were seeded in 10 cm dishes followed by 4% smoke exposure or air bolus for 6 h the following day. Media was then replaced, and the supernatant collected after 24 h was sterile filtered to produce conditioned media. Human peripheral blood mononuclear cells (PBMC) were isolated from healthy donors, treated with conditioned media for 48 h, and then harvested for analysis by flow cytometry to characterize lymphoid and myeloid immune cell phenotypes. The myeloid cell flow panel consisted of Brilliant Violet 711™ anti-mouse CD11c Antibody (117,349, BioLegend, RRID: AB_2563905), CD68-PE (333,807, BioLegend, RRID: AB_1089057), CD83-APC (305,311, BioLegend, RRID: AB_314519), HLA-DR/DP/DQ-FITC (361,705, BioLegend, RRID: AB_2563191), CD1d-BV421 (350,315, BioLegend, RRID: AB_2687378), CD80-BV605 (563,315, BD Biosciences, RRID: AB_2738135), and CD33-RB705 (757,579, BD Biosciences, RRID: AB_3662859). The lymphoid cell panel consisted of CD3-APC-Cy7 (344,817, BioLegend, RRID: AB_10644011), CD4-BV605 (300,555, BioLegend, RRID: AB_2564390), CD8a-BV711 (301,043, BioLegend, RRID: AB_11218793), CD25-AF488 (302,615, BioLegend, RRID: AB_493044), PD-1-PE (379,209, BioLegend, RRID: AB_2922607), Lag3-BV421 (369,313, BioLegend, RRID: AB_2629796) and TIM3-APC (364,803, BioLegend, RRID: AB_2910409). All flow events were acquired on an LSR II flow cytometer (BD Biosciences) and analyzed with FlowJo version 10.0.00003 for MacIntosh (Tree Star, Inc., Ashland, OR, USA).

### In vivo cigarette smoke exposure

All animal experiments were conducted in accordance with the U.S. Public Health Service Policy on the Human Care and Use of Laboratory Animals and the regulations of the Baylor College of Medicine Institutional Animal Care and Use Committee. To establish the chronic smoke in vivo model, six- to eight-week-old female C57BL/6J mice were exposed to an escalating dosage of cigarette exposure from 0.5 to 2.5 cigarettes per day for 8 weeks (exposure 5 days/week, weekends off) accomplished by using a Airdyne2000 Air Compressor. In parallel, MOC1 cells were chronically exposed to smoke before orthotopic inoculation. Following the inoculation of 200,000 cells orthotopically (tongue) in each mouse, cigarette smoke exposure continued throughout the experimental period. Tumors were measured throughout the experiment and were harvested for transcriptional analysis. Murine PBMCs were isolated using a lymphocyte separation medium (#25–072-CV, Corning) for subsequent flow analysis [41].

### Statistical analysis

Differences in means for two groups were analyzed by Student’s t test (a = 0.05) and single factor comparisons involving more than two groups were performed by a one-way analysis of variance (ANOVA), all using Graphpad Prism software v10, with a post-hoc Tukey honestly significant difference test employed for individual comparisons. Two-factor ANOVA, associated post-hoc testing, and calculation of Cohen’s effect size were performed in python using custom scripts, together with pandas, numpy, and modules for anova, posthoc, and statsmodels.

### Study approval

All animal studies contained herein were performed following approval by, and in accordance with the rules of the institutional (BCM) Institutional Animal Care and Use Committee.

## Results

### Tobacco exposure is associated with altered immunity and Nrf2 hyperactivation in multiple smoking-associated malignancies including HNSCC

**Fig. 1:**
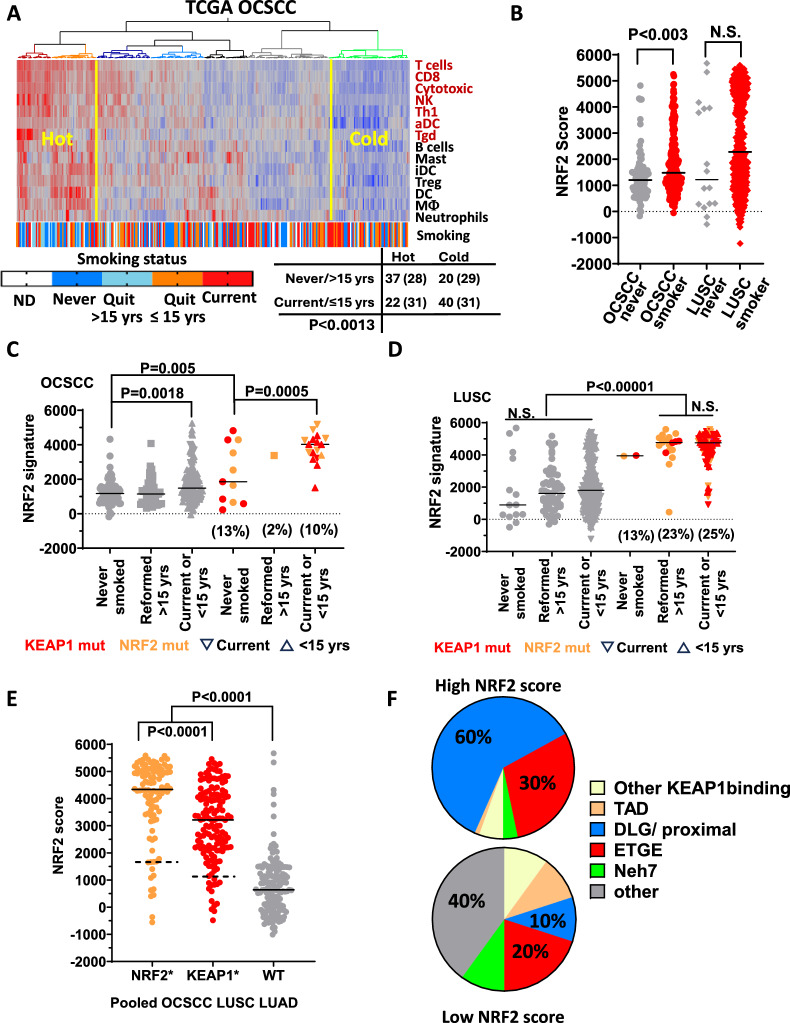
Smoking alters the tumor immune microenvironment and selects for mutations activating the NRF2 pathway. **A** Two-way hierarchical clustering for the presence of 14 different immune subtypes based on ssGSEA scores identifies a cluster of immunologically cold patient tumors from the OCSCC TCGA cohort that were enriched for patients who smoke or recently quit. The number of patients in the hot and cold clusters according to smoking status are shown in the table directly underneath, along with the numbers expected by chance if there was complete independence and the p value (p < 0.0013) obtained after χ^2^ analysis. **B** The Nrf2 pathway measured by ssGSEA scores is significantly elevated in OCSCC patients with a smoking history (p < 0.003), with a similar trend observed in LUSC tumors. **C** Stratification of patient tumors by *KEAP1* (red symbols) and *NFE2L2*(*NRF2)* (orange symbols) mutations demonstrated they were significantly associated with elevated Nrf2 pathway activation compared to mutated tumors from non-smokers in OCSCC (p = 0.0005) or patients who were wild-type (WT) for both genes (**D**), regardless of smoking history in LUSC (p < 0.0001). Both smoking and mutation were associated with elevated Nrf2 scores in OCSCC (e.g., p < 0.00001 for both variables) and showed a significant interaction (p = 0.013) with a large effect of smoking on mutation-driven Nrf2 pathway elevation (i.e., Cohen’s *d* = 1.03). **E** Pooling of TCGA data from smoking-related cancers (OCSCC, LUSC, LUAD) demonstrates that both *NFE2L2*(*NRF2)* and *KEAP1* mutations are significantly associated with elevated Nrf2 pathway activation on average compared to WT tumors, but for a minority of mutations (e.g., 10 percentile values indicated with a dashed line) the differences are marginal. **F** *NFE2L2*(*NRF2)* mutations in amino acid motifs involved in Keap1 binding (ETGE and DLG) or their immediate vicinity occurred in 90% of cases when Nrf2 scores were elevated compared to just 30% when Nrf2 activation was marginal (i.e., Nrf2 scores in the lower 10 percentile), which was found to be significant by χ^2^ analysis (*p* < 0.00001)

We previously published that overexpression of the Nrf2 downstream targets glutathione peroxidase 2 (*GPX2*) and aldo-ketoreductase family 1 (A*KR1C*) members were associated with a “cold” tumor immune microenvironment (TIME) in multiple smoking-related cancers [22] including OCSCC and non-small cell lung cancers (NSCLC). We also observed an increased frequency of *KEAP1/NFE2L2* pathway mutations in patients with cold tumors. Because *GPX2* has been described in the literature as the smoking-induced glutathione peroxidase [42], we decided to further investigate the relationship between smoke exposure, activation of the Nrf2 pathway and expression of its downstream targets, and immune phenotypes in HNSCC patient tumors and cell line models. To examine the correlation between smoking history and immune phenotypes, TCGA RNA-seq data from OCSCC patients was used to determine the distribution of tumors from individuals who smoke among patient clusters with increasing amounts of leukocyte infiltration. The ssGSEA scores from 14 different leukocyte subsets were used to cluster patients by immune profile (Fig. 1, Supplementary Table I) using methods we previously published [22]. Two adjacent clusters of patients with elevated presence of nearly every leukocyte subtype (e.g., “hot”) were easily discernable from the cold cluster with greatly reduced levels of all leukocytes. Tumors from patients who smoked concurrently with their diagnosis or had quit < 15 years before were significantly enriched among the cold tumors (Fig. 1A  p < 0.002).

Next, we investigated the relationship between smoking history and Nrf2 pathway activation in OSCC and lung squamous carcinoma (LUSC) TCGA cohorts. We previously published a gene expression signature of Nrf2 activation (e.g. score) comprised of 138 genes—many of which are known Nrf2 downstream targets—which was vetted through cross-correlation of gene expression from more than 9000 TCGA tumor samples spanning dozens of different tumor types and validated by association with the presence of *KEAP1/NFE2L2* pathway mutations [22]. There was a modest but significant increase in average NRF2 activation (p < 0.003) among OCSCC tumors from patients with a history of smoking and a trend towards increased NRF2 activation among smokers with LUSC (Fig. 1B). Patients were stratified by *KEAP1/NFE2L2* mutation status to dissect their contribution to Nrf2 activation. In OCSCC tumors, the highest Nrf2 activation was seen in smokers or those who recently quit (i.e., reformed ≤ 15 years). Although Nrf2 activation in non-smokers was higher in the presence of a *KEAP1/NFE2L2* mutation compared to non-smokers with wild-type (WT) tumors (*p* = 0.005, Fig. 1C), mutated tumors from non-smokers still had significantly lower Nrf2 scores than mutated tumors from current/recent smokers (p = 0.0005). A two-way ANOVA confirmed significant interaction between smoke and mutation status, with a large effect size (Cohen’s *d* = 1.03). Tumors from LUSC patients who smoked, regardless of when they quit, had dramatically increased Nrf2 activation compared to WT tumors regardless of smoking status (Fig. 1D). There were not enough mutated *KEAP1/NFE2L2* tumors from non-smokers to examine interaction with smoking, so we focused on whether the nature of mutations influenced their impact on NRF2 activation, after pooling TCGA data from OCSCC, LUSC, and lung adenocarcinoma (LUAD). Most *KEAP1/NFE2L2* mutant tumors had Nrf2 scores far above the average values from WT tumors (Fig. 1E), but qualitative differences were found when comparing *KEAP1/NFE2L2* mutations with Nrf2 scores in the lower 10 percentile. *NFE2L2* mutations occurring in amino acid motifs that directly bind Keap1 (i.e., DLG or ETGE) were found in 90% of tumors with higher Nrf2 activation scores compared to just 30% among mutated tumors with the lowest Nrf2 activation (*p* < 0.00001, Fig. 1F). Collectively, our data suggest that smoking selects for tumors with *KEAP1/NFE2L2* mutations, which drive increased Nrf2 activation.

### Tobacco smoke activates Nrf2 through modulation of cellular oxidative stress

**Fig. 2:**
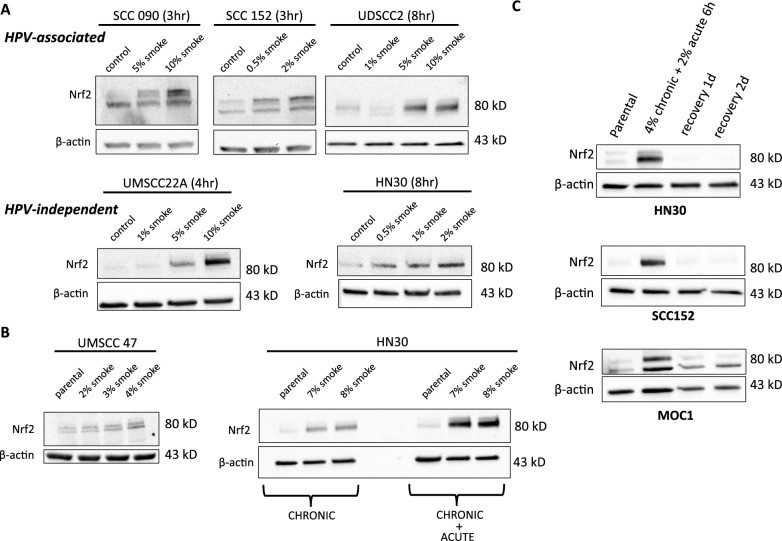
Tobacco exposure activates Nrf2 in both HPV-associated and HPV-independent HNSCC models. **A** Nrf2 protein levels increased in a dose dependent manner upon acute smoke exposure at the indicated time periods in HPV-associated (upper panel) and HPV-independent (lower panel) HNSCC cell lines. **B** Nrf2 protein levels increased in UMSCC47 and HN30 cells following chronic exposure to smoke-infused media at the indicated concentrations for 3–6 months. Right-panel lanes in HN30 cells indicate Nrf2 levels following an acute bolus of smoke 4 h before harvesting. **C** HN30, SCC152 and a murine OCSCC cell line (MOC1) were treated with an additional bolus (6 h) of smoke infused media following chronic exposure at the indicated concentrations. After removing the smoke infused media, cells were allowed to recover for 1 and 2 days prior to measurements of Nrf2 protein levels. β-actin was used as the protein loading control

**Fig. 3:**
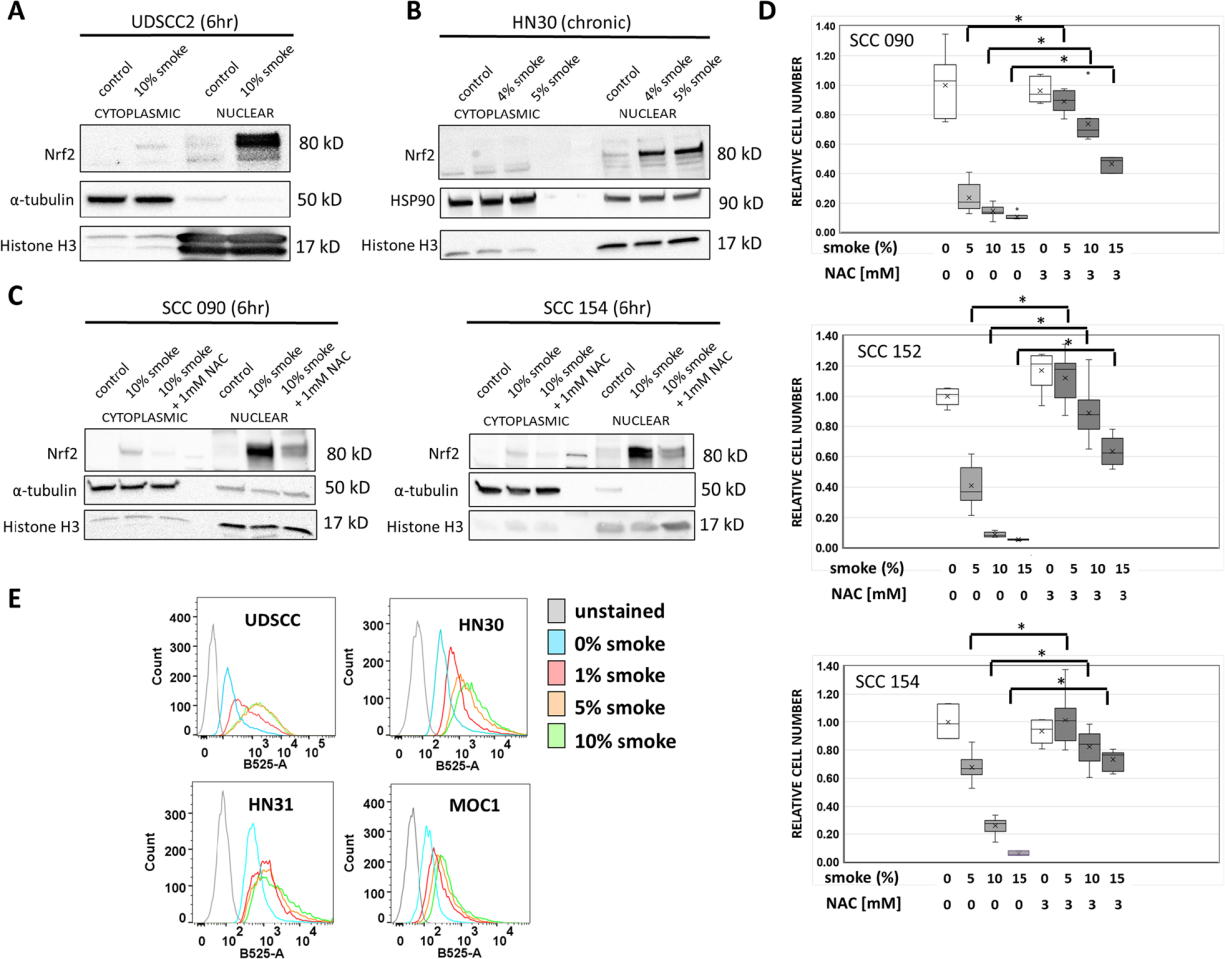
Tobacco exposure effects are ROS-mediated. Nrf2 protein levels were measured in nuclear and cytoplasm fractions after acute (6 h) smoke exposure in UDSCC2 cells (**A**) and in the chronic exposure model of HN30 cells (**B**). α-tubulin or HSP90 served as loading controls for the cytoplasmic fraction, and histone H3 for served as loading control for the nuclear fraction. **C** Pretreatment with the ROS scavenger NAC inhibited smoke-induced Nrf2 nuclear translocation following acute smoke exposure of SCC090 and SCC154 cells. **D** SCC090, SCC152, and SCC154 cells were exposed to 0%, 5%, 10%, and 15% cigarette smoke-infused media in the presence or absence of 3 mM NAC. Relative cell number at the end of a 72 h exposure period was assessed using Hoechst assay. Data are shown as means, normalized to control condition; error bars indicate standard deviation; * denotes p value < 0.05 for the compared values (smoke alone vs smoke + NAC). **E** Cellular ROS levels were detected by measuring the fluorescence of DCFH-DA (B525-A) in UDSCC2, HN30, HN31 and MOC1 cells after treatment with the indicated concentrations of smoke-infused media for 6 h

To assess whether the observed patient-level data represent simply a correlation, we conducted a mechanistic analysis of the relationship between tobacco exposure and Nrf2 pathway activation. Acute exposure to tobacco smoke led to an increase in total cellular Nrf2 protein levels in both HPV-associated (SCC090, SCC152, UDSCC2) and HPV-independent (UMSCC22A, HN30) human cell lines (Fig. 2A). *As a technical note, our conclusions regarding Nrf2 protein levels throughout these experiments were drawn from observations of the highest detected band on Western blotting, consistent with changes in the major transcript detected in HNSCC cell lines and prior literature.* Prolonged/chronic smoke exposure also resulted in elevated Nrf2 protein levels in both HPV-associated (UMSCC47) and HPV-independent (HN30) cell lines, suggesting that persistent exposure does not lead to negative feedback inhibition of the pathway (Fig. 2B). Interestingly, when acute smoke exposure was combined with chronic exposure, a very substantial increase in Nrf2 protein levels was observed (Fig. 2B). In the absence of smoke exposure, Nrf2 stabilization was reversed, and Nrf2 protein levels returned to baseline within 24 h across all tested cell lines (HN30, SCC152, MOC1) after stimulus withdrawal (Fig. 2C). Both acute (Fig. 3A) and chronic (Fig. 3B) smoke exposure induced Nrf2 localization to the nucleus in a reactive oxygen species (ROS)-dependent manner, as evidenced by the reversal of this effect upon treatment with the ROS scavenger N-acetyl cysteine (NAC) (Fig. 3C). The observed Nrf2 localization and its reversal by NAC were closely linked to the relative cytotoxicity of cigarette smoke in vitro, suggesting that Nrf2 activation functions as a survival response to exogenous ROS in HNSCC cells (Fig. 3D). This was further corroborated by direct measurements of intracellular ROS levels following cigarette smoke exposure (Fig. 3E). Exposure to smoke caused slight increases in the G2/M phase after 24 h and modest increases in both S and G2/M phase after 48 h in HN30 (Fig.S1A). This was accompanied by increased cleavage of both caspase-3 and PARP (Fig.S1B), along with a dose-dependent increase in percentage of cells staining positive for Annexin V positivity (Fig.S1C), indicative of apoptosis. Similar changes in cell cycle and dose response dependent apoptosis were also found for UDSCC2 exposed to smoke (Fig.S1D–F).

**Fig. 4:**
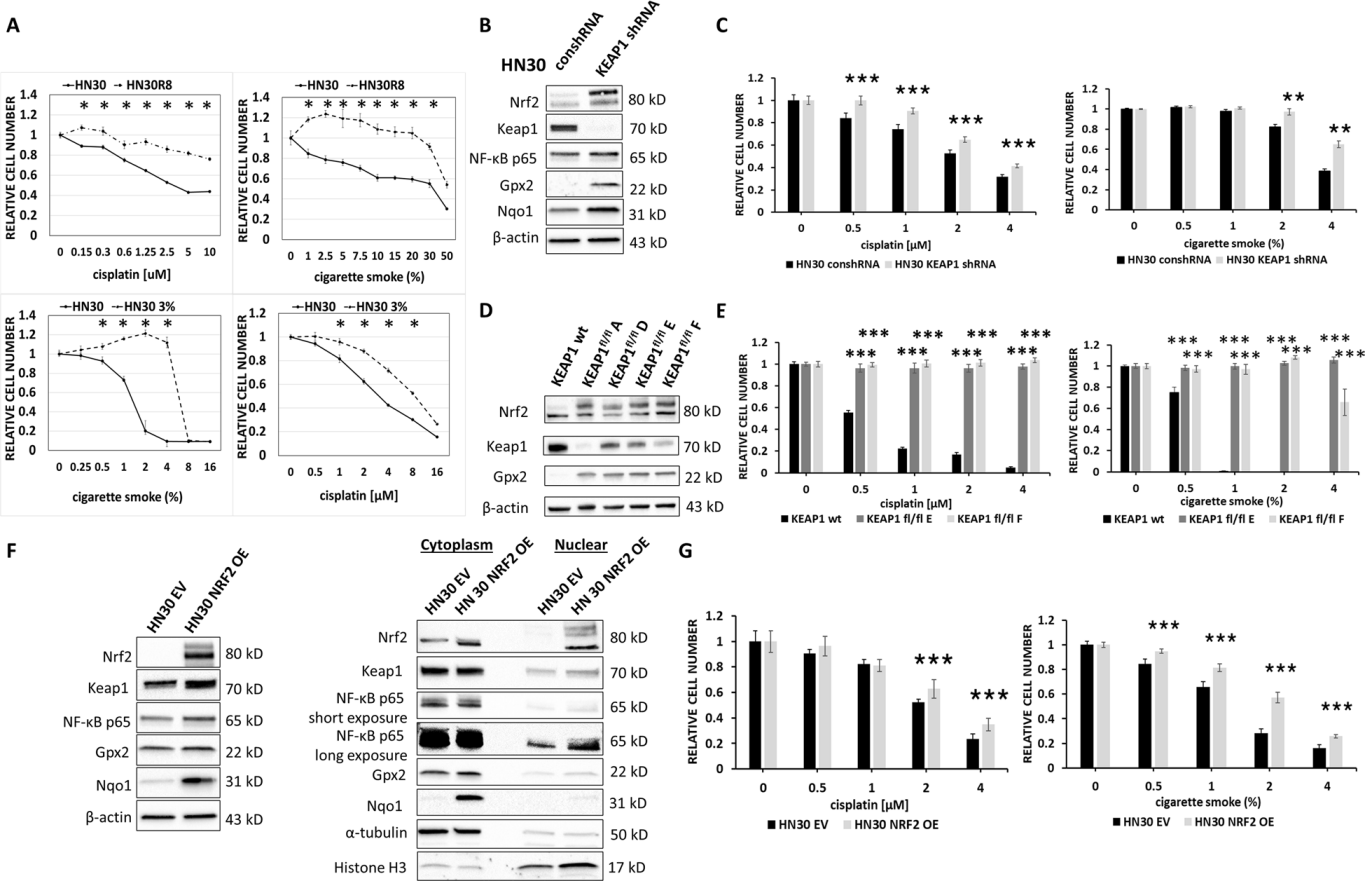
Cross-resistance to smoke and cisplatin is mediated by Nrf2. **A** Using a 72-h Hoechst assay we compared the cell number following cisplatin (left upper panel) or smoke exposure (right upper panel) in the parental HN30 cell line and its cisplatin-resistant derivative HN30R8. We then compared cell number following smoke exposure (left bottom panel) or cisplatin (right lower panel) in the parental HN30 cell line and its chronically smoke-exposed derivative HN30 3%. **B** HN30 cells were stably infected with either control shRNA or *KEAP1* shRNA resulting in increased Nrf2, Gpx2 and Nqo1 levels when Keap 1 levels were reduced. **C** HN30 *KEAP1* shRNA cells demonstrated increased resistance to both cisplatin and smoke exposure (Hoechst, 72 h). **D** *KEAP1* wild-type (wt) and *KEAP1*^fl/fl^ clones A, D, E, and F generated from HNSCC GEMM models were harvested for Nrf-2 and Gpx2 protein measurements. **E** *KEAP1*^fl/fl^ clones E and F demonstrated increased resistance to both cisplatin and smoke compared to the *KEAP1* wildtype cells (Hoechst, 72 h). **F** HN30 cells were stably infected with either human NRF2 overexpression (OE) plasmid or empty vector (EV). Whole cell lysates and cell fractions were analyzed for Nrf2, Keap1, Gpx2, Nqo1 and p65 protein levels. α-Tubulin served as loading control for the cytoplasmic fraction, and histone H3 served as loading control for the nuclear fraction. **G** HN30 cells with NRF2 OE demonstrated increased resistance to both cisplatin and smoke (Hoechst, 72 h). Hoechst data are shown as means, normalized to control condition; error bars indicate standard deviation; p values are denoted as *p < 0.05, **p < 0.01, and ***p < 0.001

We previously reported that evolution of cisplatin resistance was facilitated by Nrf2 pathway activation in multiple different HNSCC genetic backgrounds which occurred both in the presence and absence of obvious *KEAP1/NFE2L2* pathway mutations and drove an enhanced reductive metabolic state [19]. Consistent with an ROS-mediated mechanism of Nrf2 activation and cytotoxicity, we found that HNSCC cells adapted to higher levels of ROS through cisplatin exposure (HN30R8) demonstrated significant resistance to cigarette smoke (Fig. 4A, upper panels). We then performed the converse experiment, by chronically exposing HN30 to increasing concentrations of smoke-infused media, up to a maximum of 3% over a 9 month period. Chronic exposure to smoke-infused media significantly reduced sensitivity to cisplatin (Fig. 4A, lower panels).

To establish a causal link between Nrf2 activation and sensitivity to tobacco exposure in HNSCC, we used complementary molecular approaches to manipulate Nrf2 activity. Knockdown (KD) of *KEAP1* expression using shRNA increased intra-cellular Nrf2 protein levels (Fig. 4B) and reduced sensitivity to both smoke and cisplatin (Fig. 4C). The same effect was measured in the mouse cell line, MOC1 (data not shown). Because *KEAP1* loss of function mutations are a driver of HNSCC [43], we pivoted to analysis of cell lines derived from a GEMM tumor model in which a single *KEAP1* copy was deleted. We analyzed levels of Nrf2 and its downstream target Gpx2 in *KEAP1*^fl/fl^ clones derived from GEMM tumors and found stable and persistently high levels of Nrf2 and Gpx2 protein (Fig. 4D), which correlated with profound resistance to both smoke and cisplatin (Fig. 4E). Additionally, we overexpressed (OE) human *NFE2L2* (i.e., Nrf2) in the HPV-independent HN30 cell line, increasing Nrf2 protein levels, translocation into the nucleus and increasing protein levels of the Nrf2 targets Gpx2 and Nqo1 (Fig. 4F). *NFE2L2* OE conferred increased resistance to smoke and cisplatin treatments (Fig. 4G). It is important to note that, although molecular manipulation of *NRF2* or *KEAP1* can impact the phenotypic effects of smoke and cisplatin exposure, both *KEAP1* OE and *NFE2L2* OE or KD lose penetrance over prolonged cell passaging resulting in loss of phenotype (data not shown).

### Nrf2 activation by smoke generates an enhanced reductive state

**Fig. 5:**
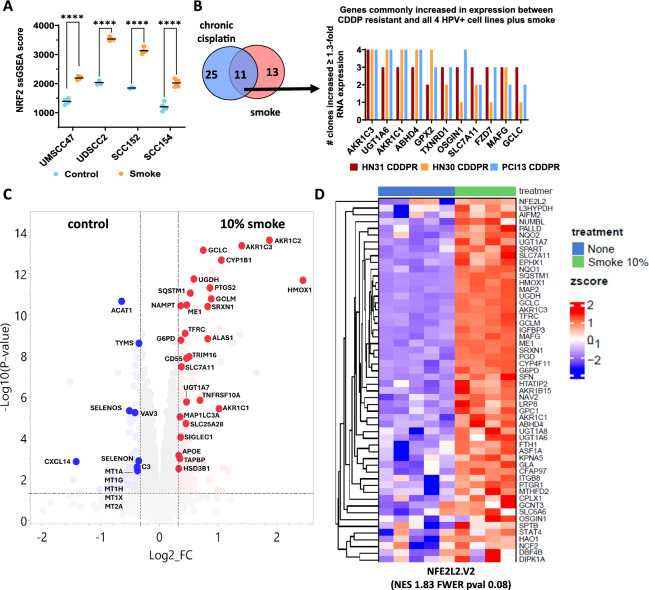
Smoke exposure activates Nrf2 targets consistently upregulated in cisplatin resistant HNSCC. **A** NRF2 ssGSEA scores are significantly higher in both smoke-naïve (UMSCC47 and UDSCC2) and chronically exposed cell lines (SCC152 and SCC154) following an 8 h bolus of acute smoke. **** adj p value < 0.0001. **B** Venn diagram illustrating the number of NRF2 signature genes significantly elevated in 12 clones derived from 3 different cisplatin (CDDP)-resistant HNSCC cell line genetic backgrounds, 4 HPV-associated HNSCC cell lines treated with smoke, and their overlap. A total of 36 common NRF2-target genes were significantly elevated in clones from all 3 different CDDP resistant cell lines (HN30, HN31, and PCI13) and 11 of these genes (listed to the right) overlapped with all 4 HPV-associated HNSCC cell lines exposed to smoke. **C** Volcano plot of UDSCC2 proteins as a function of fold change and log10 p value at 10% smoke exposure generated using JMP Pro version 17 (SAS Institute Inc., 2023). A cutoff of 1.25 fold change (equivalent to 0.322 log2 fold change) and a false discovery rate (FDR) of 0.05 (equivalent to -log10(p value) of 1.30) were applied. The y-axis represents -log10 p value, while the x-axis displays log2 fold change. Targets associated with metabolism, immunity, and Nrf2 signaling are highlighted. Downregulated targets are indicated in blue, and upregulated targets are shown in red. **D** Heatmap of proteins significantly elevated (FDR = 0.1) by smoke exposure (10%) from the Molecular Signature Database NRF2 pathway (i.e., NFE2L2.V2), which were identified by mass spectrometry

To measure downstream cigarette smoke effects on Nrf2 target genes, we exposed two smoke-naïve HPV-associated HNSCC cell lines (UMSCC47 and UDSCC2) and two chronically smoke-exposed HPV-associated HNSCC cell lines (SCC152 and SCC154) to an acute bolus of 4% smoked media for 8 h. A total of 309 genes were significantly upregulated by ≥ 1.3-fold in three or more cell lines exposed to smoke, which included 53 Nrf2 downstream targets (Supplementary Table II) and 256 genes not directly linked to Nrf2 signaling (Supplementary Table III). There were 344 genes commonly downregulated in 3 or more cell lines by ≥ 1.3-fold following smoke exposure (Supplementary Table III), and only two of those were in the Nrf2 pathway (Supplementary Table II). In all four lines, smoke exposure dramatically increased Nrf2 pathway activation scores (ssGSEA) (*p* < 0.0001, Fig. 5A), consistent with our western blot results demonstrating increased levels of total cellular or nuclear Nrf2 protein (Fig. 3). In all, we found 81 Nrf2 target genes significantly upregulated by smoke in at least one of the cell lines, compared to only 30 Nrf2 target genes downregulated in at least one cell line, consistent with strong Nrf2 activation.

The top Nrf2 targets upregulated by smoke *across all four cell lines* included 24 genes commonly increased by smoke ≥ 1.3-fold (Supplementary Table IV), with *AKR1C1, AKR1C3, GCLM, GCLC,* and *GPX2* among the top 10, showing average fold change increases in expression from 3 to 9. Among the commonly upregulated Nrf2 targets, *AKR1C1, AKR1C3*, and *ALDH3A2* all function in lipid peroxide detoxification while *GPX2* reduces hydrogen peroxides as well as fatty acid hydroperoxides. Also universally elevated were *SLC7A11, GCLM, GCLC,* and *CHAC1* which are all functionally linked to maintaining glutathione levels and the antioxidant *SRXN1* (Supplementary Table III). Suppressors of ferroptosis (*STC2*) and NF-κB levels (*SQSTM1*) were also among Nrf2 signature genes elevated following smoke exposure (Supplementary Table III) along with the autophagy-related gene *OSGIN1* (Supplementary Table III). A total of 11 Nrf2 targets were significantly elevated in cisplatin resistant cells from all three genetic backgrounds and in all four HPV-associated HNSCC cell lines stimulated by smoke (Fig. 5B). These Nrf2 targets included genes functioning in lipid peroxide detoxification (*AKR1C1, AKR1C3, GPX2*), glutathione and redox homeostasis (*GCLC, SLC7A11, TXNRD1*), autophagy (*OSGIN1*), *WNT* signaling (*FZD7*), and xenobiotic detoxification (*UGT1A6*).

We identified 46 genes that were not downstream of Nrf2 but were upregulated by ≥ 1.3-fold in all 4 cell lines treated with smoke (Supplementary Table V). These genes were significantly enriched for regulation of cell proliferation, protein metabolism, apoptosis and response to stress (Supplementary Table VI). Among the Nrf2-independent genes commonly upregulated we identified: (1) *CDKN1A*/p21 which mediates cell growth arrest; (2) heat shock proteins *HSPA1A* and *HSPA1B* which mediate cell survival to stress; and (3) *PIM1* and *PIM3*, two pro-survival kinases that inhibit apoptosis. A total of 76 genes were significantly decreased by ≥ 1.3-fold in all HPV-associated cell lines after smoke treatment (Supplementary Table V) and these were enriched for genes regulating nucleosome organization, mucosal immune response, and innate immune response in mucosa (Supplementary Table VI)—all of which were driven by downregulation of various histone genes. Among these were *H2BC10*,11,12, and 12L which have been directly linked with innate immunity [44]. In all, there were roughly 40 different histone proteins downregulated in all lines following smoke treatment (Supplementary Table VI), although *pathways* tied to proliferation were noticeably absent.

To validate the transcriptomic data, we performed mass spectrometry-based proteomic analysis of UDSCC2 cells acutely exposed to either 5% or 10% cigarette smoke-infused media and confirmed that the Nrf2 signaling pathway was profoundly enriched (Fig. 5C, D), particularly those proteins essential to the oxidative stress response. This was followed by the P53DN.V1 pathway consistent with the expected inactive functional status of p53 in a HPV-associated HNSCC cell line (Supplemental Table VII). A similar analysis with SCC152 chronically exposed to smoke and restimulated with an acute bolus of smoke identified the Nrf2 pathway among the most upregulated. These cells also showed upregulation of the G2M checkpoint (Hallmark pathway)―consistent with a shift from rapid proliferation toward enhanced biomass generation―allowing for survival under stress conditions as we previously demonstrated in the context of cisplatin (Fig.S2; Supplemental Table VIII).

### Metabolomic shifts following smoke exposure support an enhanced reductive state

**Fig. 6:**
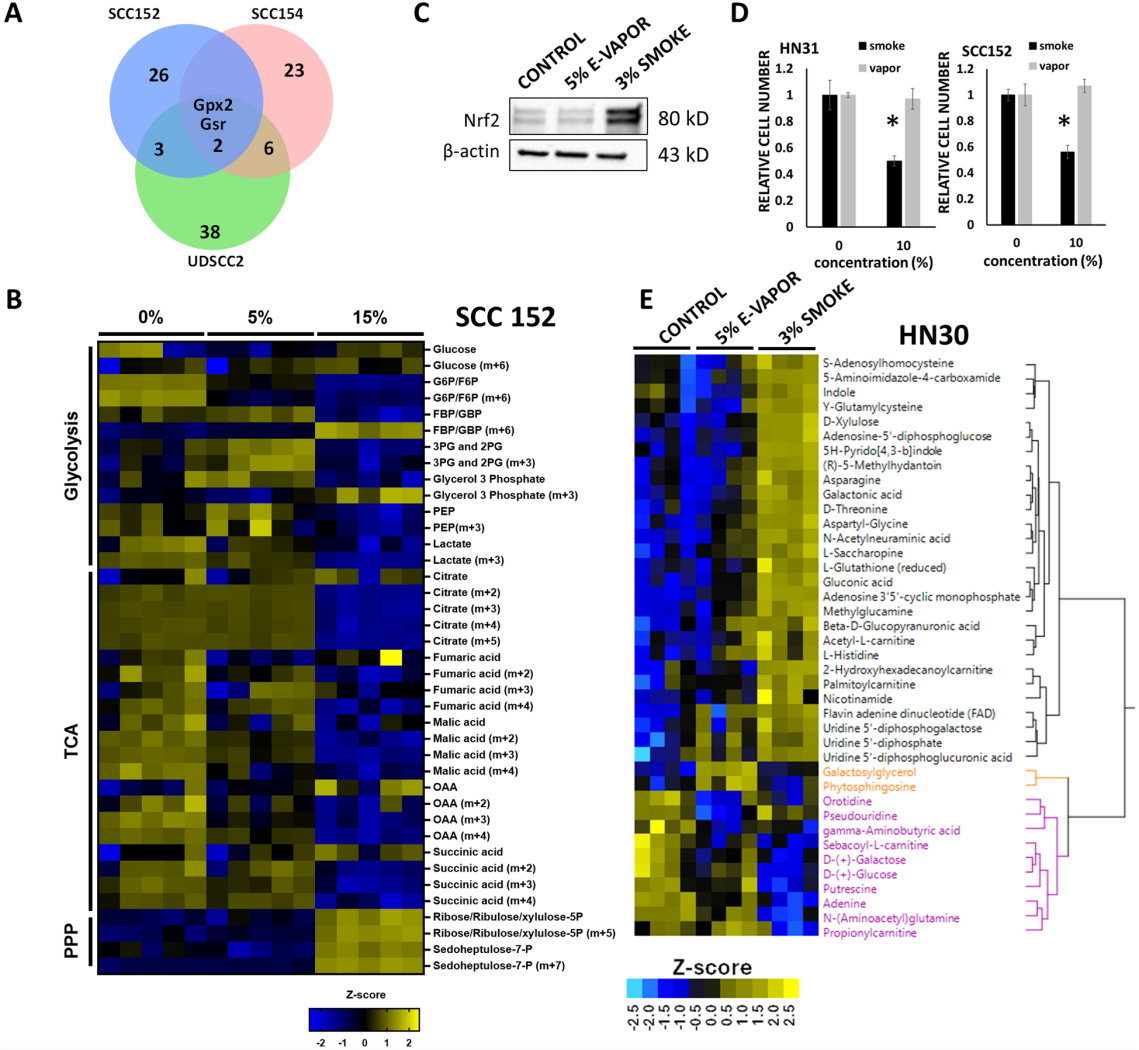
Smoke but not e-cigarette vapor shifts metabolism toward an enhanced reductive state. **A** Metabolomic and transcriptomic data were integrated across 3 distinct cell backgrounds (SCC152, SCC154, UDSCC2) and genes which were consistently upregulated (FDR ≤ 0.05) across all 3 backgrounds, defined within both datasets were identified: Gpx2 (glutathione peroxidase 2), Gsr (glutathione reductase). **B** SCC152 cells were exposed to smoke (0%, 5%, 15%) for 8 h in the presence of 25 mM ^13^C all labeled glucose (Glc). Following completion of exposure, cell lysates were analyzed for ^13^C incorporation which demonstrated reduced flux toward lactate and TCA with enhanced flux into PPP intermediates. **C** HN30 cells were exposed to smoke (3%) or e-cigarette vapor (5%) for 8 h. Smoke but not vapor increased Nrf2 total protein levels; β-actin was used as the protein loading control. **D** In contrast to smoke, vapor generated minimal effects on tumor cell viability (HN31, SCC152) even when adjustments were made for similar nicotine delivery levels (Hoechst, 72 h). Hoechst data are shown as means, normalized to control condition; error bars indicate standard error of the mean; p values are denoted as *p < 0.05. **E** In parallel (to experiment carried out in panel **C**, cells were subjected to unbiased steady state metabolomics analysis which demonstrated that smoke, but not vapor shifts tumor metabolism toward an enhanced reductive state. Two-way unsupervised hierarchical clustering of non-exposome metabolites and cell lines illustrating major differences between cells exposed to smoke and minor differences in cells exposed to vapor. *Note: Panel E and Figure S3 summarize different aspects of the same experiment*

Steady-state metabolomic analysis of UDSCC2 (Supplemental Table IX), SCC152 (Supplemental Table X), and SCC154 (Supplemental Table XI) revealed a shift in metabolite levels consistent with an altered reductive state. This included elevated levels of NAD, homocysteine, glutathione, as well as increased 3-carbon glycolytic intermediates such as glycerol-3-phosphate and phosphoenolpyruvate. These changes echoed the transient and permanent alterations in the reductive state we previously linked to cisplatin induced oxidative stress [19, 45, 46] and include metabolites involved directly or indirectly in reducing-oxidative (REDOX) carbon flux reactions of primary metabolic pathways [19, 45, 46]. Additionally, carnitine derivatives, including butyryl carnitine and isobutyryl carnitine, were elevated following smoke exposure, suggesting activation of the bi-directional carnitine shuttle, which is essential for balancing glycolytic and fatty acid metabolism [47]. To gain further insight into the most significant metabolic effects of smoke exposure across different cellular backgrounds, we integrated steady-state metabolomic data with transcriptomic data using a previously described methodology [36, 37]. As summarized in Supplemental Table XII- UDSCC (FDR = 0.05), Supplemental Table XIII-SCC152 (FDR = 0.05), Supplemental Table XIV- SCC154 (FDR = 0.05), the greatest impact of tobacco exposure occurred in pathways specifically designed to restore the cellular reductive state through recycling of glutathione and other primary and secondary reducing equivalents. Of note, the 2 enzymes consistently identified across all tested backgrounds were Gpx2 and Gsr (glutathione-disulfide reductase) (Fig. 6A). Collectively, these findings strongly suggest that tobacco exposure induces a metabolic shift aimed at enhancing the cellular reductive state by redirecting carbon flux to support the generation and/or recycling of reducing equivalents.

To demonstrate that smoke exposure actually impacted carbon flux, we measured the flux from ^13^C isotopically labeled glucose toward glycolytic, tricyclic acid (TCA) and pentose phosphate pathway (PPP) intermediates in SCC152 and UDSCC2 (Supplemental Tables XV, XVI). Smoke exposure reduced incorporation of ^13^C label in TCA intermediates, increased incorporation of label into the 2 PPP intermediates analyzed and shunted carbon away from glycolytic flux culminating in lactate production toward glycerol-3-phosphate and fructose 1,6-bisphophate (Fig. 6B). In contrast to carbon from glucose, carbon from ^13^C-cysteine was not differentially shunted under conditions of smoke exposure (data not shown).

E-cigarette vapor, a volatile, should deliver similar levels of nicotine, but potentially reduced levels of oxidative stress compared to cigarette smoke. Consistent with this hypothesis, e-cigarette vapor failed to activate Nrf2 (Fig. 6C). Consistent with lower levels of Nrf2 activation vapor demonstrated no significant impact on HNSCC cell viability (Fig. 6D). We next used unbiased metabolomics to determine whether smoke exposure was unique in shifting HNSCC metabolism toward an enhanced reductive state (Supplemental Tables XVII). This analysis confirmed a shift in the cellular reductive state in response to cigarette smoke but not vapor, inclusive of reduced glutathione (Fig. 6E; Supplemental Table XVIII) and gamma-glutamylcysteine, its immediate precursor. To validate that vapor exposure was in fact delivering the calculated levels of nicotine to HNSCC cells, we used the unbiased metabolomics analysis to evaluate exposome related metabolites in control, vapor and cigarette smoke exposed conditions (Fig.S3). Vapor delivered the highest measurable nicotine levels among tested conditions and shared some exposome related metabolites with conventional cigarette smoke including octyl hydrogen phthalate, phthalic anhydride and 5’-phosphoribosyl-N-formylglycinamide (Fig.S3, Supplemental Table XVIII). Levels of the oxidants 1,3-dioxolane and 1,3-dioxole were detectable in both tested conditions, although higher following smoke exposure. Overall, cigarette smoke demonstrated a substantially larger number of exposome related metabolites compared to e-cigarette vapor that were significantly increased compared to the control condition.

### Nrf2 activation and immune modulation

**Fig. 7:**
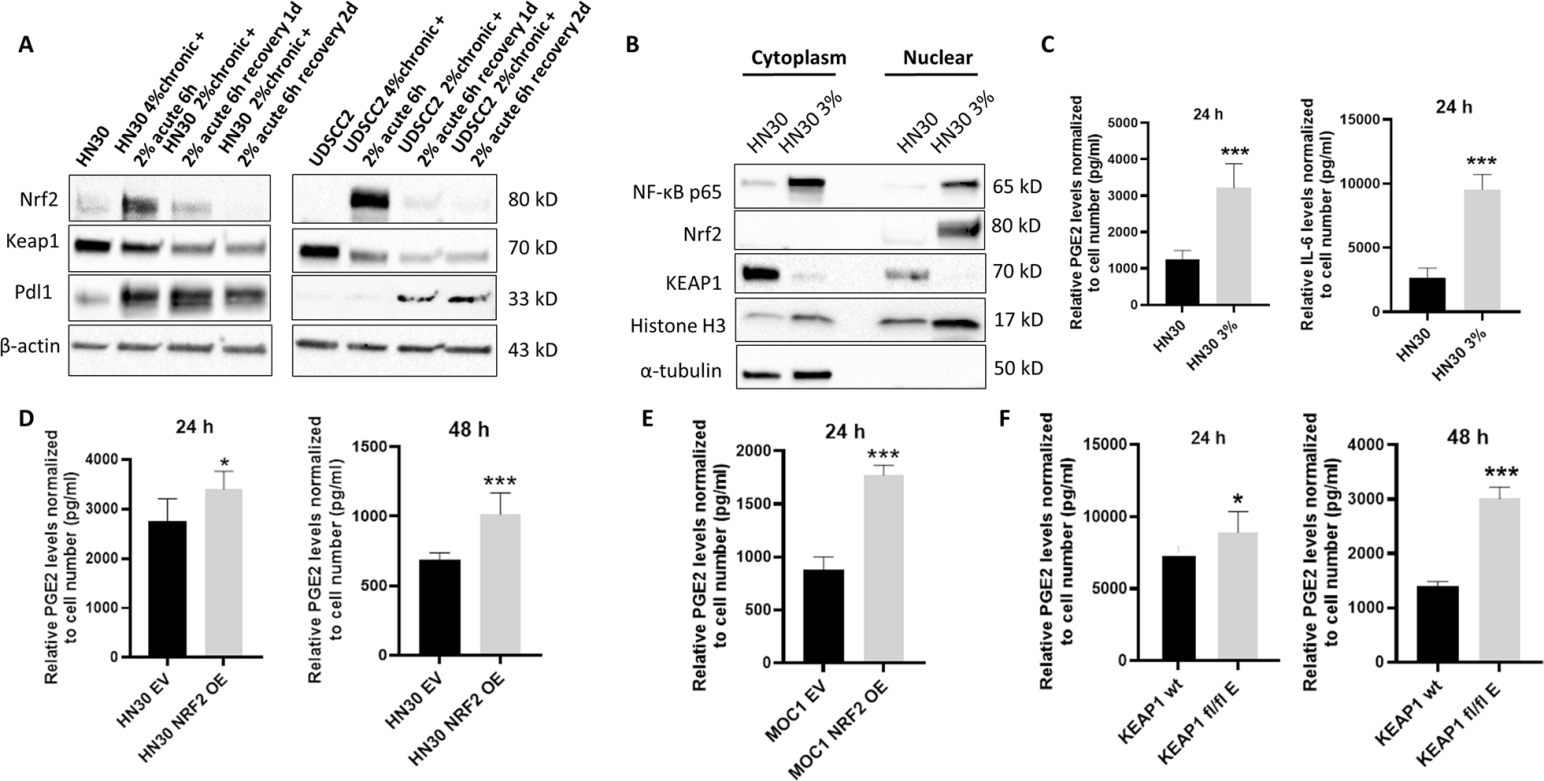
Nrf2 activation alters PDL1 expression and the secretory phenotype of HNSCC cells. **A** UDSCC2 and HN30 cells were chronically exposed to cigarette-infused media at specified concentrations for 3–6 months. An additional bolus of 2% smoke was administered for 6 h, followed by a change to fresh media for 24 and 48 h before harvesting for western blot analysis. β-actin was used as the protein loading control. **B** HN30 cells, chronically exposed to 3% smoke infused media demonstrated higher protein levels of Nrf2 and p65. **C** These cells secreted higher levels of PGE2 and IL-6 measured via ELISA. **D** HN30 cells stably overexpressing (OE) NRF2 secreted higher levels of PGE2 (collected over 24 and 48 h) compared to HN30 cells stably overexpressing empty vector (EV). **E** MOC1 cells stably overexpressing NRF2 secreted higher levels of PGE2 (collected over 24 h) compared to MOC1 cells stably overexpressing empty vector (EV). **F** *KEAP1* fl/fl clone E secreted higher levels of PGE2 (collected over 24 and 48 h) compared to its *KEAP1* wild-type (wt) counterpart. ELISA data are shown as means, normalized to control condition; error bars indicate standard deviation; p values are denoted as *p < 0.05, **p < 0.01, and ***p < 0.001

Given the shifts in gene expression following Nrf2 activation by smoke exposure, we sought to measure its impact on inflammatory mediator production and immune cell function, in vitro, in vivo and using conditioned media approaches. As shown in Fig. 7A, a bolus of smoke exposure transiently activated Nrf2 expression in chronic smoke-exposed HN30 and UDSCC2 cells. However, Pdl1 levels were substantially increased in both cell lines even after Nrf2 protein levels returned to baseline. In chronically smoke-exposed HN30 cells (Fig. 7B), the accumulation of NF-κB in the nucleus corelated with increased production of prostaglandin E2 (PGE2) and interleukin-6 (IL-6) (Fig. 7C). In contrast, acute smoke exposure led to elevated Nrf2 with a reduction in PGE2 levels for both HN30 and UDSCC2, that were consistent with failure of NF-κB translocation to the nucleus (Fig.S4). Collectively, the data show that nuclear accumulation of NF-κB is likely driven by chronic cellular stress and correlated with increased PGE2 and IL-6 secretion; whereas Nrf2 likely counters this effect in a more acute setting. In the absence of smoke bolus, ectopic expression of Nrf2 led to increased PGE2 secretion in both HN30 (Fig. 7D) and MOC1 cells (Fig. 7E). Elevated Nrf2 protein levels and PGE2 secretion were also mirrored in the *KEAP1* knockout GEMM cell lines (Fig. 7F).

**Fig. 8:**
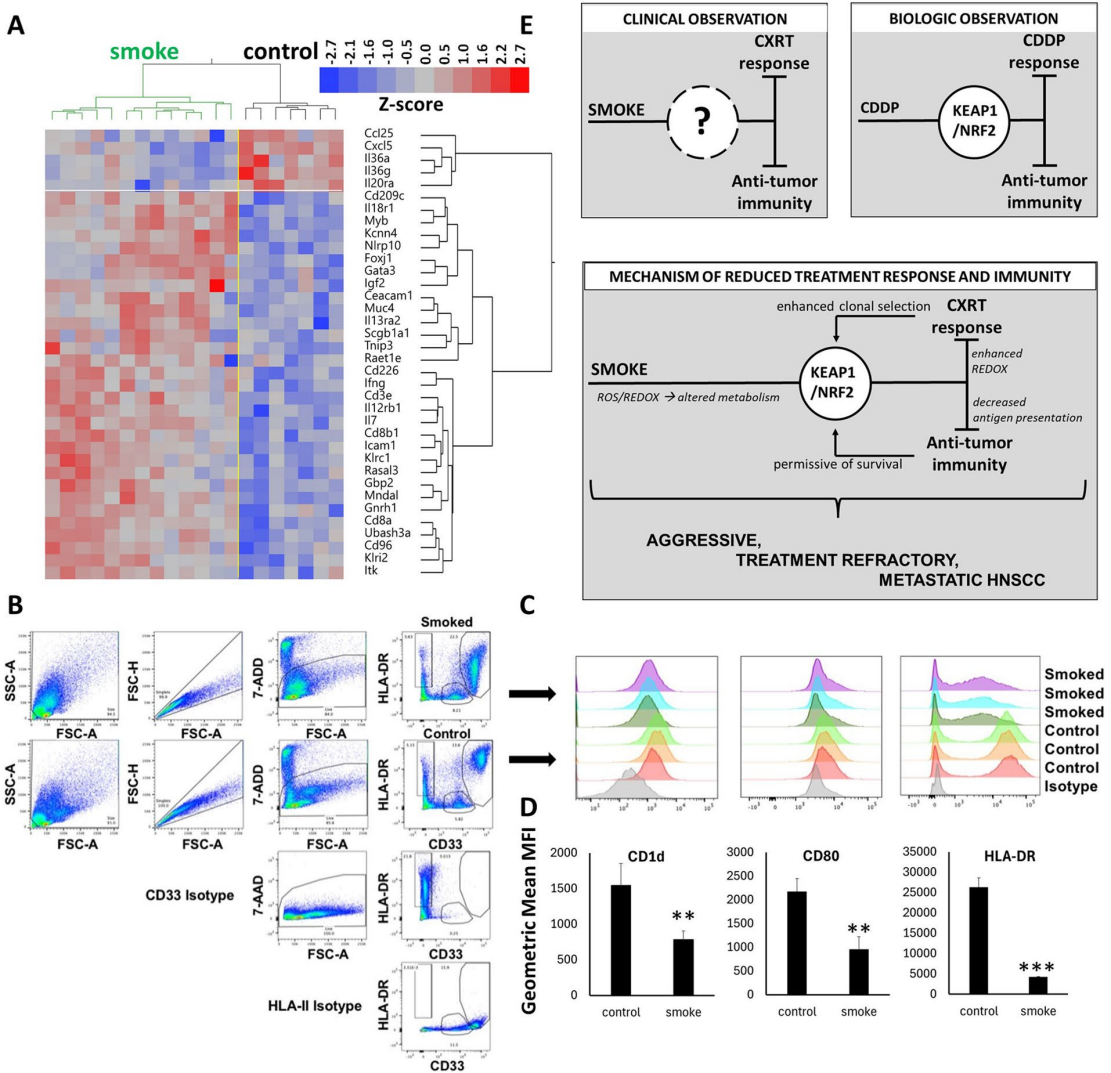
Smoked exposure modulates anti-tumor immunity. **A** MOC1 cells were used to generate orthotopic tongue xenografts in immunocompetent mice. MOC1 cells chronically exposed to 4% smoke were used to generate orthotopic tongue xenografts in chronically smoke exposed immunocompetent mice. Tumors were harvested, snap frozen and subjected to RNAseq analysis. HN30 tumor cells exposed to tobacco smoke chronically (4%) followed by an acute bolus (4%) and HN30 cells exposed to control air-infused media were used to generate conditioned media for 24 h. Cell culture media was harvested and added to PBMC derived from three healthy donors which were cultured for an additional 48 h. Cells were then harvested and characterized by flow cytometry. **B** Myeloid cell gating strategy. **C** Flow cytometry histograms of antigen presentation marker expression CD1d, CD80, and HLA-DR. **D** Graphical quantitation of flow histograms shown in C; **p < 0.005, ***p < 0.001 by Student’s paired two-tailed t test. **E** Mechanistic model of reduced treatment response and immunity in chronic smokers with HNSCC. Clinical studies have definitively linked tobacco smoke exposure to reduced chemo-radiation (CXRT) response and altered immunity in HNSCC. Pre-clinical models of HNSCC have shown that hyper-activation of the Nrf2 pathway can drive resistance to cisplatin (CDDP) and alter the inflammatory profile of HNSCC cells and tumors. The current work proposes that the exposome, specifically smoke exposure activates a self-re-enforcing loop whereby activation of Nrf2 predisposes HNSCC tumors to reduced treatment response and suppressed immunity, generating an aggressive, treatment refractory and highly metastatic phenotype

We performed duplicate in vivo experiments to recapitulate chronic smoke exposure using the murine oral cancer cell line MOC1 [48], which was implanted orthotopically in syngeneic immunocompetent mice. MOC1 cells, exposed to cigarette smoke (final concentration, 4%) for a period of > 4 months were inoculated into mice that had also been chronically exposed to cigarette smoke and the smoke exposure was continued in vivo until completion of the experiment. Control MOC1 cells, never exposed to smoke were inoculated in mice with no cigarette smoke exposure. Variable rates of tumor growth were noted, with no clear change in tumor growth velocity attributable to smoke exposure. To determine whether the chronic smoke exposure altered the intrinsic biology of the tumors or the tumor immune microenvironment, RNAseq was used to compare 8 control tumors and 12 smoke exposed tumors. We identified 187 mouse genes significantly increased by ≥ 1.5-fold in smoke exposed tumors, compared to 203 genes downregulated by ≥ 1.5-fold (Supplementary Table XIX) from smoke. Genes upregulated in smoke exposed tumors mapped to GO pathways related to: (1) negative T cell selection, (2) regulation of NK cell mediated immunity and (3) negative regulation of lymphocyte mediated immunity among others (Supplemental Table XX). In contrast, downregulated genes were enriched for just one GO pathway, cell adhesion (*P* = 0.006). Among the significantly altered genes we identified 31 upregulated that were linked to Go immune pathways (Fig. 8A), which included T-cell receptor components (*CD3E*, *CD8A*), a few cytokine receptors, interferon gamma, and IL-17. In contrast, we observed decreased levels of chemokines *Cxcl5* (neutrophil recruitment) and *Ccl25* (T-cell recruitment). Although up-regulation of *NFE2L2* itself was not detectable under these in vivo conditions, there was significant up-regulation of *NFE2L3* and the downstream target *Akr1C18* (Supplemental Table XIX) which has been previously linked to prostaglandin metabolism.

Given the difficulty of capturing the complete human smoking phenotype using a murine model, we used a simpler conditioned media exposure model to ascertain the effects of smoke exposure on the interaction between HNSCC cells and PBMCs. Two different approaches were utilized. First, bulk RNAseq was used to examine changes in the transcriptional signature of PBMCs following incubation with conditioned media that had been collected from HNSCC cells previously treated with a bolus of smoke followed by replacement with fresh media. As summarized in Supplemental Table XXI, slightly over 200 genes were significantly upregulated by > twofold in PBMCs incubated with smoke exposed conditioned media (i.e., SECM) but many more genes (i.e., > 800) were downregulated by this level. GO pathways enriched (Supplemental Table XXII) among these down-regulated genes included: (1) antigen processing and presentation, (2) MHC protein complex assembly and (3) positive regulation of immune effector process among others. Multiple chemokine genes and their receptors were downregulated along with nine different MHC class II genes (Supplemental Table XXIII). Upregulated genes were enriched for pathways regulating leukocyte chemotaxis, B cell and mononuclear cell differentiation (Supplemental Table XXII), which included cytokines and other inflammatory genes (Supplemental Table XXIII).

In a second approach, flow cytometry was used to characterize shifts in PBMC fate triggered by incubation with SECM. SECM treatment led to significant shifts in PBMC differentiation as shown in Fig. 8B, C and Supplemental Table XXIV, which included dramatically lower expression of MHC class II only within the myeloid CD11c^+^ (and not CD19^+^, not shown) antigen presenting cell population, substantially lower CD80 (costimulatory) and CD1d (lipid antigen presentation) expression levels (Fig. 8D).

## Discussion

Oxidative stress management plays a crucial role in both tumor formation and the effectiveness of anticancer treatments [49]. Historically, Nrf2 was thought to primarily function as a tumor suppressor by regulating antioxidant gene expression and detoxifying reactive oxygen species (ROS) [50]. However, recent studies have challenged this perspective using new evidence. ROS and the metabolic adaptations which accompany ROS exposure, have now been shown to mediate cellular reprogramming by selecting for aggressive features, such as enhanced invasiveness and resistance to therapy. We recently linked hyperactivation of Nrf2 through both mutational and transcriptional mechanisms to acquisition and maintenance of resistance to chemotherapy and radiation, the two mainstay treatments for HNSCC as well as for many other solid tumors [18, 19, 51]. Whereas selection of inactivating *KEAP1* or activating *NFE2L2* mutations in the context of treatment-induced oxidative stress can be detected in both preclinical models and human tumors, there remains an important question, namely whether some tumors are predisposed to hyperactivation of this pathway and development of subsequent treatment resistance.

Over the last two decades, we and others have conclusively shown that smokers, particularly active smokers demonstrate inferior responses to chemoradiotherapy, regardless of HPV-association, resulting in higher rates of disease recurrence and cancer specific death [3, 11]. In parallel, we measured a distinct tumor immune microenvironment using overlapping approaches, strongly suggestive of suppressed anti-tumor immunity, presumably explaining a more aggressive clinical phenotype [10, 52]. The current study sought to bring together these two observations into one comprehensive model, congruent with clinical reality. The model, outlined in Fig. 8E highlights an iterative process by which the smoke exposome predisposes a subset of HNSCC tumors to hyperactivation of the Nrf2 signaling pathway which is then exacerbated by initiation of chemoradiotherapy. Persistent and successful Nrf2 hyperactivation can then drive a dual phenotype of enhanced survival under conditions of ROS mediated stress through metabolic reprogramming and an altered secretory phenotype which can generate immune-suppressive effects.

As shown here, Keap1-Nrf2, although an exquisitely sensitive ROS sensor, and an adaptive mechanism for treatment resistance, can be maintained in a relatively narrow range as demonstrated by the difficulties associated with exogenous molecular manipulation of either *KEAP1* or *NFE2L2* (in contrast to routine manipulation of other oncogenes and tumor suppressors such as *TP53*, *PIK3CA*, *NOTCH1*). This raises the critical question of whether primary tumors that successfully evolve with *KEAP1/NFE2L2* mutations necessitate the co-evolution of as-yet-unidentified permissive pathways, which might be leveraged for targeted therapy. Together with evidence that the activation pattern of Nrf2 is rather short-lived but can be re-activated by repeated exposure, we can now understand a manner by which this pathway is persistently activated through chronic, daily exposure to cigarette smoke (and other environment toxins) leading to selection for cells and tumors predisposed toward chemoradiotherapy resistance. That persistent smoke exposure can directly lead to acquired cisplatin resistance is shown here directly as is the cross-resistance of cisplatin resistant cells to the oxidative stress generated by cigarette smoke. Notably, although e-cigarette vapor can deliver substantial nicotine levels to HNSCC cells, in contrast to conventional cigarette smoke, the oxidative stress component and the metabolic disruptions associated with the reductive state appear to be significantly diminished if not completely absent. Not only are cigarette smoke effects on activation of the Nrf2 pathway consistent across HNSCC cell lines of variable genomic background and HPV-association, but the effects are clearly coordinated at transcriptional, translational and metabolic levels, with overall organization of the response aimed at enhancing the response to exogenous oxidative stress.

Nrf2-hyperactivated human tumors have been shown by us and others to demonstrate an altered tumor immune microenvironment (TIME), with significant indications of suppressed, or inactive anti-tumor immunity [22]. Existing TCGA data clearly show a strong correlation between tobacco exposure, Nrf2 activation, and altered immunity. Here we present a rather comprehensive mechanistic analysis of this interaction and demonstrate that exposure of HNSCC to tobacco changes Pdl1 levels. However, the combined impact on Nrf2 and NF-κB is more nuanced, and likely reflects the balance of cellular reducing potential. It is widely known that ROS triggers both Nrf2 and NF-kB**,** which have antagonistic functions [53]. Elevated ROS drive NF-kB activity leading to increased inflammatory mediators, whereas downstream targets of Nrf2 reduce ROS to potentially inhibit NF-κB and shut down inflammation. Since both pathways are simultaneously triggered by cigarette smoke, the final phenotype depends on their balance which is intimately linked to exposure levels, timing, and REDOX homeostasis―all of which can be difficult to experimentally model. In light of our previous work connecting Nrf2 activation to an immunosuppressive tumor immune microenvironment for smoking related cancers, it is likely that this pathway dominates these tumors over the long run. Regardless, there is a clear connection between cellular insults and the inflammatory secretome of tumors. This was made apparent by changes in the inflammatory mediators PGE2 and IL-6 secreted by smoke exposed cells, through experiments showing conditioned media from smoke exposed tumor cells can rewire PBMC programing and differentiation, and by changes in the tumor immune microenvironment we found in vivo with smoke exposure.

The dual role of Nrf2 hyperactivation on intrinsic chemoradiation responsiveness and altered anti-tumor immunity has significant translational implications. Current algorithms for HNSCC rely heavily on immune checkpoint inhibitors (ICIs) in the recurrent/metastatic setting [54]. More recent trials have sought to combine ICIs with conventional chemoradiation strategies with very limited success [55]. The data summarized here and our previous mechanistic studies suggest that ICIs are likely to have limited effectiveness in Nrf2 hyperactivated tumors as might be encountered in a population enriched for heavy smokers. Furthermore, as cisplatin and radiation—based treatment are likely to further select for tumors with extremely high levels of activation[18, 19, 23], ICI-based rescue of conventional treatment failure, or potentiation of conventional chemoradiation strategies may very well be unfeasible at least in a subset of HNSCC [56]. Ongoing studies in our group, utilizing non-invasive imaging of the tumor reductive state, circulating biomarkers of Nrf2 hyperactivation and spatial transcriptomic analysis of primary and recurrent HNSCC tumors are attempting to define the clinical window in which activation of this pathway is expected to generate a maximal deleterious effect on both conventional and ICI-based treatment efficacy.

Our data support the translational conclusion that Nrf2 activation represents a functional nexus between metabolic reprogramming designed to support oxidative stress, development of treatment resistance and altered immunity. Several questions remain unanswered. First, what is the role of nicotine, exclusive of oxidative stress? Nicotine has been shown to stimulate cancer progression mediated by the activation of nicotinic acetylcholine receptors (nAChRs) [57]. This signaling has been linked to promoting lung carcinogenesis and facilitating immune evasion by smoke-induced macrophages in NSCLC cells [58]. Similar research also suggests that activating nAChR7 contributes to immune evasion triggered by cigarette smoke, by increasing Pdl1 levels in lung epithelial cells [59]. Although lacking an oxidative stress component, e-cigarette vapor exposure will require more extensive in vivo modelling to determine whether the immunomodulatory effects we capture with conventional cigarette smoke are at least partially replicated in the absence of the oxidative volatile agents.

Second, although we have identified what we believe are some of the critical components of the Nrf2 pathway related to oxidative stress response and altered metabolism both here and in our previous publications [18, 19, 23, 45], we found numerous Nrf2 target genes are robustly upregulated by smoke. Others have reported that Nrf2 activation can regulate the formation of stress granules which mediates selective translation of genes important for survival of cancer cells under conditions of genotoxic stress [60]. Focused screens using either shRNA or CRISPR will be required to determine how many and which down-stream effectors are both necessary *and* sufficient to support the reductive state adaptation described here and the acquisition of chemoradiotherapy resistance and altered immunity.

Although we use multiple overlapping models of HNSCC and recapitulate much of the HNSCC biology encountered in the clinical setting (*TP53* wild type *vs* mutant, HPV-associated *vs* HPV-independent) we are limited in our ability to precisely model human tobacco exposure in murine models with respect to both time and dose. As a result, we rely heavily on in vitro modeling which omits many of the feedback loops and multi-cell compartment regulatory mechanisms that exist in vivo. Modelling the precise interaction between Nrf2 and NF-kB in particular will require development of inducible knockout and overexpression vectors coupled with additional experiments in the orthotopic HNSCC model. Finally, newly developed GEMM models, which are only partially leveraged here for their stable Keap1 suppressed status, will require further examination to better understand the effects of chronic cigarette smoke exposure and development of chemoradiotherapy sensitivity and/or resistance. By studying chronic smoke exposure in GEMM models, in which the tumor and the TIME co-evolve, we believe it may be possible to identify those critical transition points at which anti-tumor immunity begins to fail in its containment function and the degree to which smoke exposure can impact the process.

## Supplementary Information

Additional file1

Additional file2

## Data Availability

The data generated in this study are available within the article and its supplementary files.
