# Supplementary material for: Tobacco smoke exposure is a driver of altered oxidative stress response and immunity in head and neck cancer
Source: J Transl Med. 2025 Apr 5;23:403. doi: 10.1186/s12967-025-06258-z (PMC11971752; doi:10.1186/s12967-025-06258-z)
Supplement: Supplementary file 1 — Additional file1 [file 12967_2025_6258_MOESM1_ESM.pdf]

SUPPLEMENTAL FIGURE S1

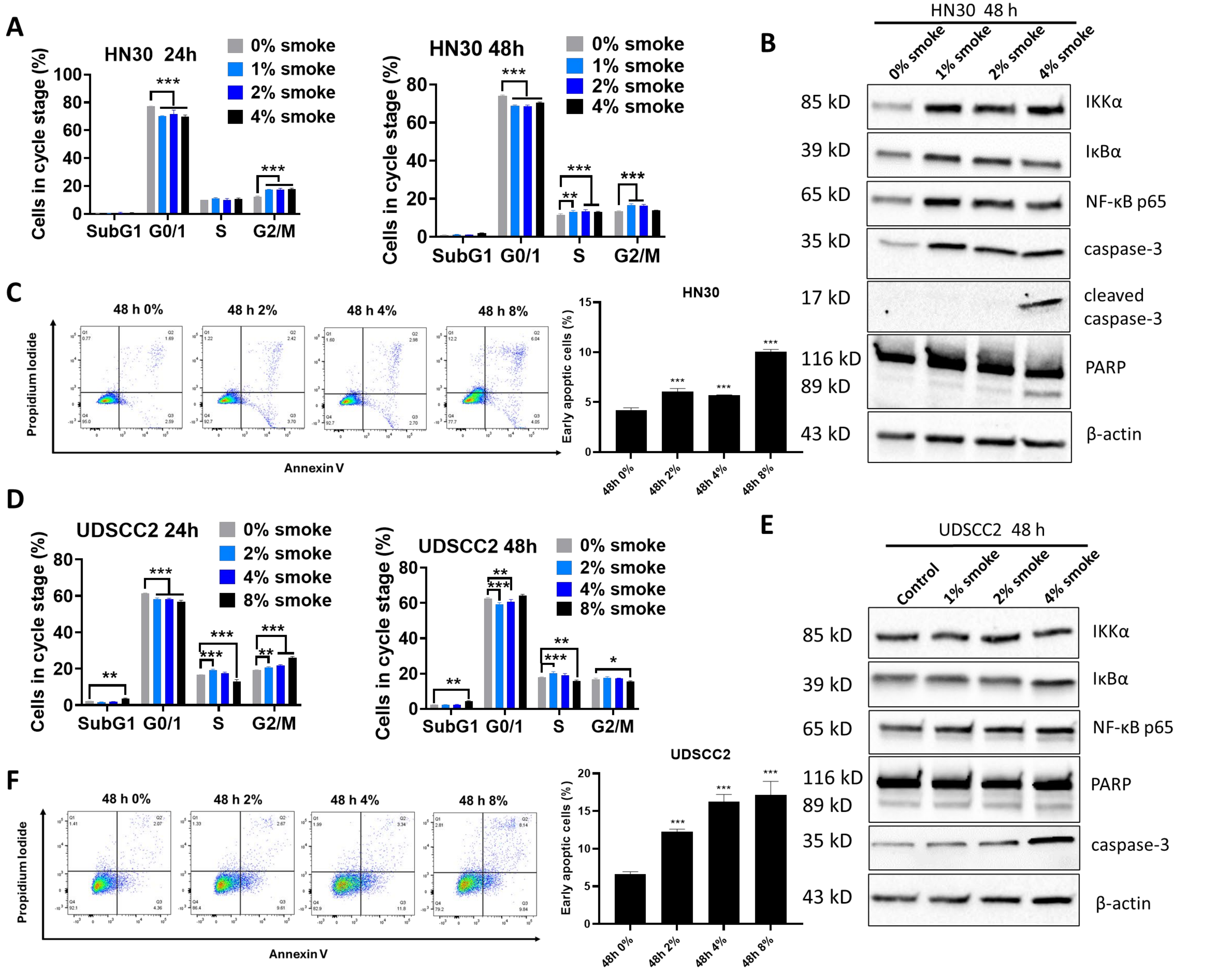

Supplemental Figure 1. Tobacco exposure induces cell death in HNSCC. Propidium iodide (PI) staining was employed to evaluate the impact of smoke on cell cycle progression in HN30 (A) and UDSCC2 (D) cells. PI combined with Annexin V staining was performed to examine the effects of smoke on early apoptosis of UDSCC2 (C) and HN30 (F) cells. Western blot analysis of HN30 (B) and UDSCC2 (E) cells at 48 hours post-smoke exposure was conducted to assess NF-kB activation along with PARP and caspase-3 cleavage. Data are shown as means, normalized to control condition; error bars indicate standard deviation; p-value s are denoted as *p<0.05, **p<0.05, and ***p<0.001.

SUPPLEMENTAL FIGURE S2

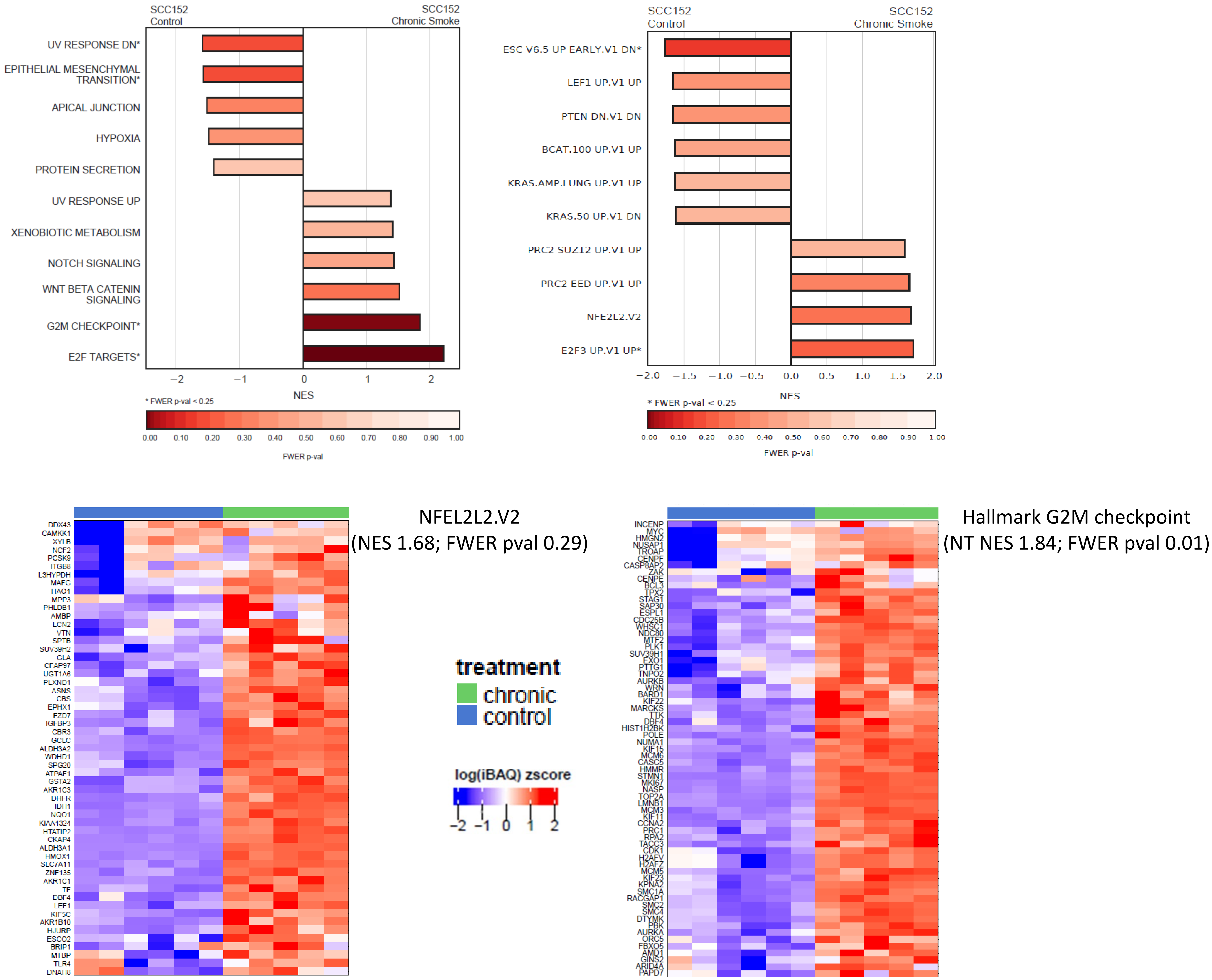

Supplemental Figure 2. Chronic smoke exposure activates Nrf2 dependent protein synthesis and reduces proliferation. SCC152 cells were chronically exposed to 5% cigarette smoke infused media and exposed to an acute bolus of 5% smoke infused media; proteins were isolated 12 hours following the acute exposure. Pathway enrichment analysis using the Hallmark (left) and Oncogenic 6 (right) panels. Nrf2 and G2M checkpoint pathway protein levels are illustrated below, left and right respectively.

SUPPLEMENTAL FIGURE S3

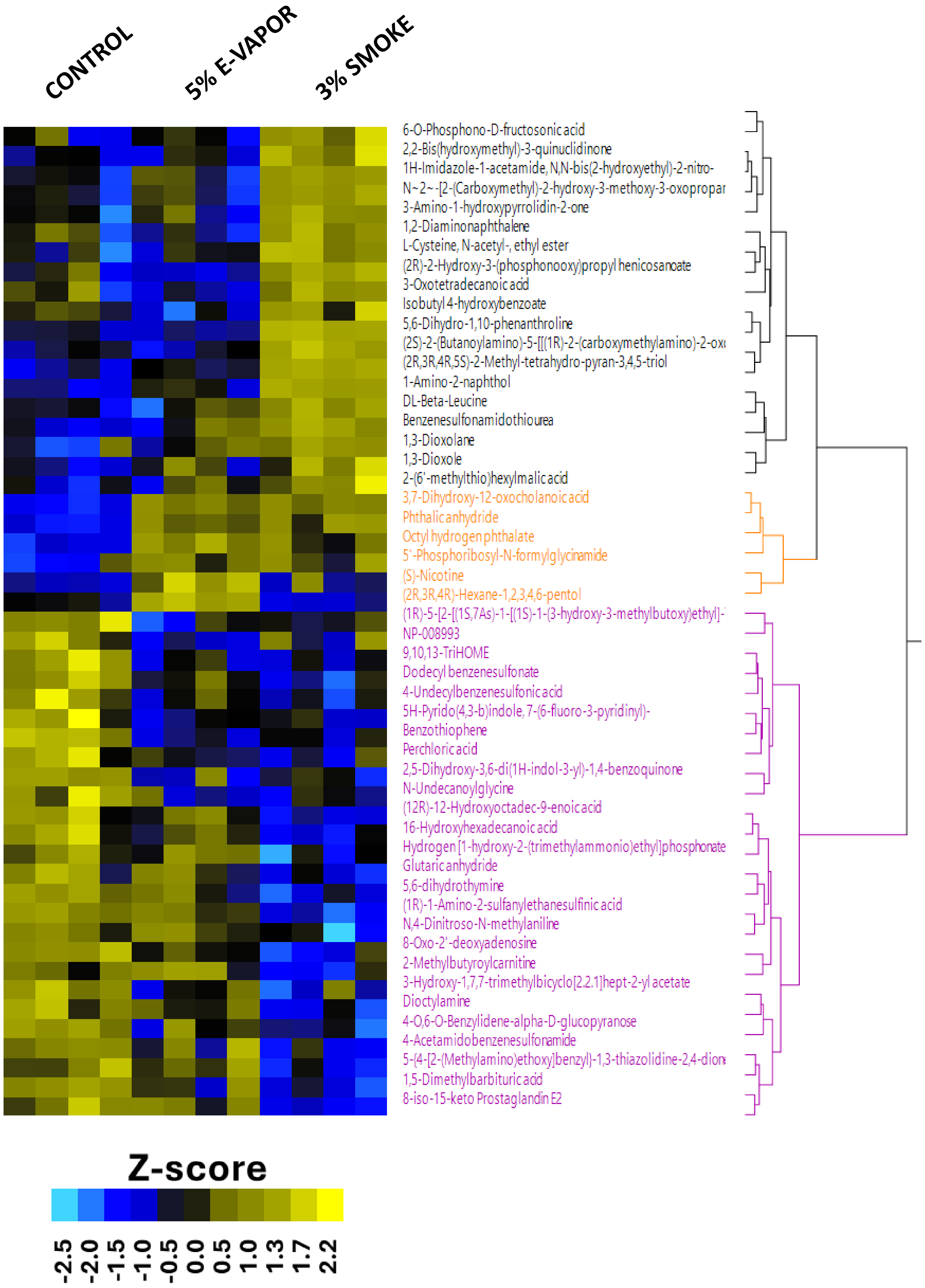

Supplemental Figure 3. Differential signature of exposome metabolites following smoke and vapor exposure. HN30 cells were exposed to smoke or vapor for 8 hours. Exposome-related metabolites were analyzed and compared among the groups using unbiased steady state metabolomics.

SUPPLEMENTAL FIGURE S4

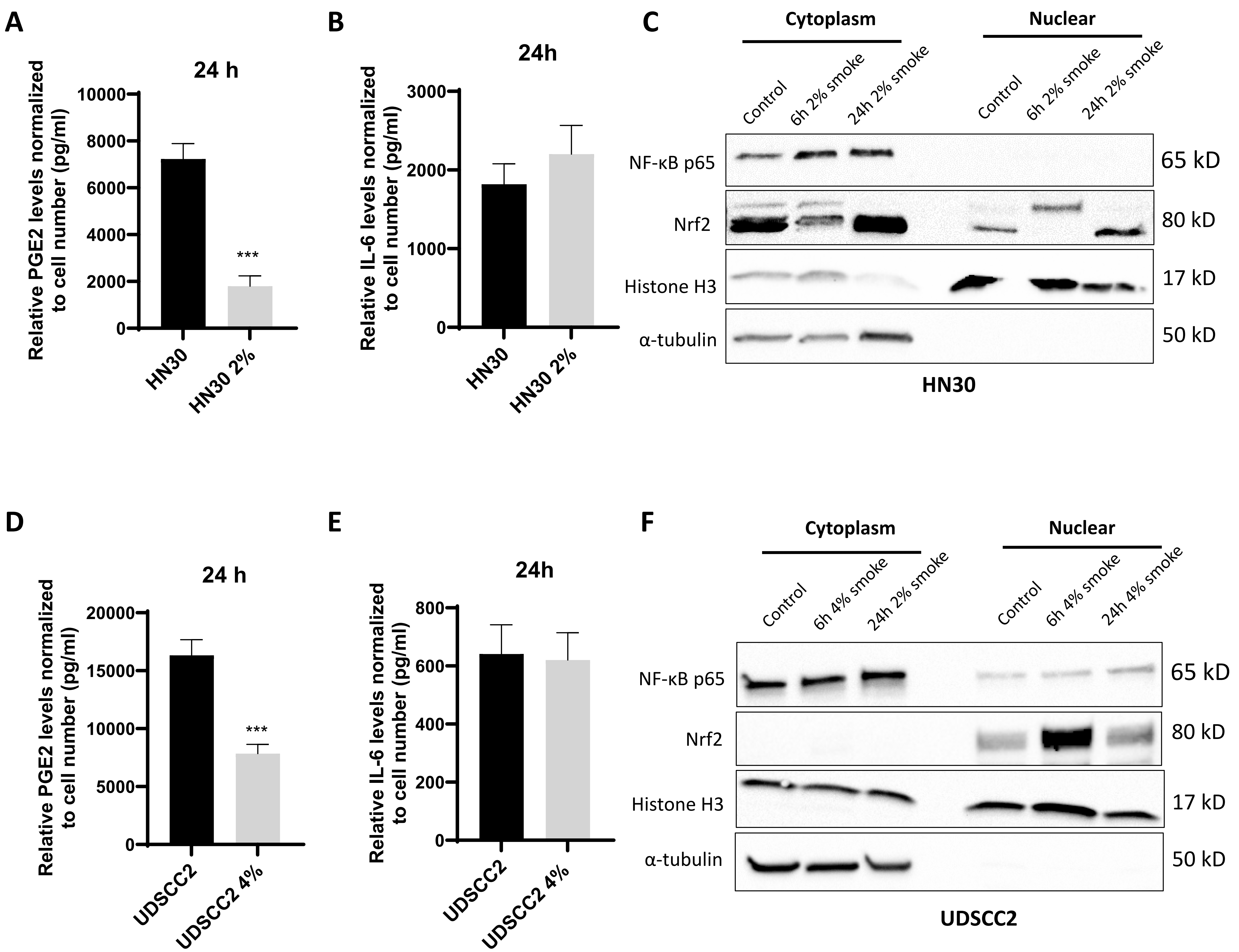

Supplemental Figure 4. Acute smoke exposure and immune modulation. The corresponding cells were seeded at the same density on day 1 and treated with or without 2% smoke media the following day (day 2). Then the conditioned media were harvested after 24 h (day 3), and the total number of cells counted accordingly. Secreted PGE2 (A, D) and IL-6 (B, D) levels were measured using the corresponding ELISA kit and normalized to cell number. All data are repeated in triplicates and represented as mean ± SD. p-values were calculated using Student's t-test. (*P<0.05, **P<0.05, and ***p<0.001). (C, F) Fractionation was conducted to confirm the translocation of Nrf-2 targeted proteins. α-Tubulin served as loading control for the cytoplasmic fraction, and histone H3 served as loading control for the nuclear fraction. *p<0.05, **P<0.05, and ***p<0.001
